# Brain Neural Progenitors are New Predictive Biomarkers for Breast Cancer Hormonotherapy

**DOI:** 10.1158/2767-9764.CRC-21-0090

**Published:** 2022-08-24

**Authors:** Agnes Basseville, Chiara Cordier, Fadoua Ben Azzouz, Wilfried Gouraud, Hamza Lasla, Fabien Panloup, Mario Campone, Pascal Jézéquel

**Affiliations:** 1 Omics Data Science Unit, Institut de Cancérologie de l'Ouest (ICO), Angers-Nantes, France.; 2 SIRIC ILIAD, Angers-Nantes, France.; 3 Laboratoire Angevin de Recherche en Mathématiques (LAREMA), Université d'Angers, Angers, France.; 4 Institut de Cancérologie de l'Ouest (ICO), Angers-Nantes, France.; 5 CRCI2NA, Inserm UMR1307/CNRS UMR 6075/Université de Nantes, Nantes, France.

## Abstract

Heterogeneity of the tumor microenvironment (TME) is one of the major causes of treatment resistance in breast cancer. Among TME components, nervous system role in clinical outcome has been underestimated. Identifying neuronal signatures associated with treatment response will help to characterize neuronal influence on tumor progression and identify new treatment targets. The search for hormonotherapy-predictive biomarkers was implemented by supervised machine learning (ML) analysis on merged transcriptomics datasets from public databases. ML-derived genes were investigated by pathway enrichment analysis, and potential gene signatures were curated by removing the variables that were not strictly nervous system specific. The predictive and prognostic abilities of the generated signatures were examined by Cox models, in the initial cohort and seven external cohorts. Generated signature performances were compared with 14 other published signatures, in both the initial and external cohorts. Underlying biological mechanisms were explored using deconvolution tools (CIBERSORTx and xCell). Our pipeline generated two nervous system-related signatures of 24 genes and 97 genes (NervSign24 and NervSign97). These signatures were prognostic and hormonotherapy-predictive, but not chemotherapy-predictive. When comparing their predictive performance with 14 published risk signatures in six hormonotherapy-treated cohorts, NervSign97 and NervSign24 were the two best performers. Pathway enrichment score and deconvolution analysis identified brain neural progenitor presence and perineural invasion as nervous system-related mechanisms positively associated with NervSign97 and poor clinical prognosis in hormonotherapy-treated patients. Transcriptomic profiling has identified two nervous system–related signatures that were validated in clinical samples as hormonotherapy-predictive signatures, meriting further exploration of neuronal component involvement in tumor progression.

**Significance:**

The development of personalized and precision medicine is the future of cancer therapy. With only two gene expression signatures approved by FDA for breast cancer, we are in need of new ones that can reliably stratify patients for optimal treatment. This study provides two hormonotherapy-predictive and prognostic signatures that are related to nervous system in TME. It highlights tumor neuronal components as potential new targets for breast cancer therapy.

## Introduction

Breast cancer is a leading cause of cancer death worldwide. In Europe, it caused about 138,000 deaths in 2018. This high death rate is mainly due to treatment resistance in metastatic breast cancers. The 5-year survival rate for women with metastatic breast cancer is 27%, whereas it is 90% for all combined breast cancers (1, 2). Even if 6%–10% of breast cancers are already metastatic when they are first diagnosed, most of them are recurrences from initial cancers. To reduce breast cancer recurrence, adjuvant therapy is almost systematically administrated postsurgery, with a choice of radiotherapy, hormonotherapy, chemotherapy, and/or targeted therapy (3).

Adjuvant therapy is chosen according to clinical characteristics (lymph node status, tumor size, and tumor grade), patient physiologic status and age, and IHC status in three tumor receptors: estrogen receptor (ER), progesterone receptor, and HER2. In addition to these clinicopathologic factors, and although they have no decisional value on their own, six genomic signatures are currently available in clinic (all marketed, two FDA approved) to assist oncologist in optimal therapeutic choices: Mammaprint, OncotypeDX, 76-gene Rotterdam signature, GGI-97, Prosigna PAM50, and Endopredict (4). All are prognostic signatures (two of them are also chemotherapy-predictive), mainly aimed at ER-positive patients and all include a subset of genes that are proliferation markers. Determining breast cancer subtypes and relapse risk—and tailoring treatment accordingly—has led to increased survival for breast cancer patients over the last decades (3). Challenges remain for patients classified with intermediate risk of relapse for whom therapeutic decision is always delicate, and for patients with high-risk relapse, for whom new therapeutic targets are needed. In their cases, both early and late relapses still occur, highlighting unfulfilled medical needs.

One of the emergent leading causes of treatment failure is tumor heterogeneity. Indeed, tumor is not a homogeneous entity to treat, but a complex association between cancer cell subclonal populations—driven by their own genetic alterations—and the tumor microenvironment (TME) including immune cells, stromal cells (like fibroblasts, adipocytes, vascular system and neuronal cells) and the extracellular matrix (ECM). In fact, TME is inextricably linked to cancer progression, from *in situ* early lesion to local invasion and migration (5). It regulates processes necessary for tumor development such as restructuration of ECM (permitting to isolate the nascent tumor), evolution of immunity to a more permissive environment, or angiogenesis/lymphangiogenesis and neurogenesis/axonogenesis necessary for its proliferation and dissemination (6). Over the past decade, TME has been constantly pointed out for its significant role in resistance to treatment. Several components of microenvironment have already been clinically associated with breast cancer recurrence, such as subsets of cancer-associated fibroblasts (CAF; ref. 7), tumor-infiltrating lymphocytes (8), angiogenesis (9) or elements of the ECM (10). The relationship between TME neuronal components and breast cancer treatment outcome were also investigated, but the few studies conducted were not always concordant (11, 12) or based on very small patient cohorts (13). In fact, among all TME components, the biology behind the nervous system is one of the least understood. Only recently, a rising interest in cancer neurobiology suggested its underestimated role in cancer recurrence, advocating for deeper investigations (14).

One way to decipher a TME component association with clinical response is targeted biomarker investigation from massive “omics” data from patients. To proceed, datasets are explored by combining mathematical and biological approaches (15). Machine learning (ML) is first used to extract new variables thanks to its high potential to explore complex and multivariate biological data. Next, variable analysis based on prior biology knowledge helps to decipher new leads, confounding variables, or to exclude already known mechanisms. Finally, the newly discovered TME markers are tested for their capacity to stratify patients according to treatment outcome.

In this study, we used ML combined with literature-based analyses to explore nervous system involvement in breast cancer resistance. Our pipeline allowed the identification of early-stage neural progenitors as new hallmark of cancer recurrence for hormonotherapy treatment, and the derivation of two predictive gene expression signatures (GES) from TME neuronal components.

## Materials and Methods

All analysis were performed using R Project for Statistical Computing (RRID:SCR_001905, version 4.0.3). The used packages are indicated in italic, and referenced in Supplementary Table S1.

### Patient Cohort

Retrospective, nonblinded analysis was performed on patients with breast cancer from stage IA to IIIC (invasive nonmetastatic cancers). Patient cohort was created using publicly available datasets for which transcriptomics analysis of the tumor was performed prior to any treatment, and relapse status is known. bc-GenExMiner v4.5 online tool (16) was used as repertoire to select clinically annotated datasets that will be merged. Patient characteristics and inclusion criteria are detailed in Supplementary Table S2. Because of the lack of publicly available RNA sequencing (RNA-seq) dataset associated with relapse response information, only microarray-derived transcriptomic were used. To decrease bias effect due to manufacturer, we selected microarray datasets from Affymetrix platform only, with 10,000 quantified genes and 50 patients at least. Raw data were normalized using RMA (*simpleaffy*). To combine datasets, probes were transformed to HUGO gene symbol, aggregated by median, and reduced to 12,659 genes that are shared across all Affymetrix platforms. Three cross-platform normalization algorithms were tested [remove batch effect (*limma*), ComBat (*sva*), quantile normalization (*preprocessCore*)], and the best performer was selected according to performance estimated by gPCA (*gPCA*), batch correlation with principal component (*FactoMineR*), and Kolmogorov Smirnov test (*BEclear*) on gene distribution.

After cross-platform normalization, ML analysis was performed on transcriptomics data for patients that received hormonotherapy and for whom relapse status was known. Survival analysis was performed on patients for whom relapse-free survival (RFS) was known. Because sample size was not chosen by rational but determined by public dataset availability, seven external validation cohorts (described below) were used to assess the prognostic value of the GES.

### Validation Cohorts

Buffa cohort (Illumina microarray), Chanrion cohort (noncommercial microarray), and Saal cohort (Illumina RNA-seq) were downloaded from Gene Expression Omnibus (GEO). Guedj cohort (Affymetrix) was used in the initial cohort for normalization, but was not included in patient selection due to lack of individual clinical information: the nonnormalized dataset was therefore used as external validation. Juin cohort (Affymetrix) is composed of patients with breast cancer treated at the Institut de Cancérologie de l'Ouest (ICO), for whom surgery was performed between 2009 and 2016, and follow-up was done until 2021. The transcriptomic data were previously published and deposed on GEO (17). METABRIC dataset (Illumina) was downloaded from cBioPortal. ICO triple-negative breast cancer (TNBC) cohort are patients with TNBC treated at the ICO for whom transcriptomic analysis of the tumors was performed prior to treatment (18), as well as IHC for UCHL1/PGP9.5 and S100 neurologic markers (19). Patient characteristics for each validation cohort are detailed in Supplementary Table S3.

RFS was the clinical endpoint used for all datasets but Saal dataset, for whom overall survival (OS) was used.

### Class Balancing by Resampling

Four methods were assessed to oversample the minority class: SMOTE (synthetic minority oversampling technique), ADASYN (adaptive synthetic sampling approach for imbalanced learning), Bordeline-SMOTE and relocating safe-level SMOTE (*smotefamily*). Two methods were used to undersample the majority class: randomly picked sample (with conservation of batch proportion) and cluster centroids (k-means based). Efficiency of the methods were tested by comparing random forest prediction performance (*randomForest*) on the majority and minority classes (sensitivity and specificity, respectively) using 5-fold cross-validation (*caret*).

### Response Prediction and First Signature Generation

Eight ML-based models were developed to predict relapse in patients using elastic net (*glmnet*), K nearest neighbors (*rknn*), logistic regression, neural network (*nnet*), random forest, SVM (*kernlab*), VSURF (*VSURF*), and XGBoost (*xgboost*) algorithms. For each algorithm, five conditions of variable (gene) selection (*t* test based) were assessed. When possible, grid search was performed for each algorithm/variable selection combination to identify the best tuning hyperparameters (*caret*). Tested parameters are presented in Supplementary Table S4. Model performances were estimated using Matthews correlation coefficient (MCC). For each model, the best combination algorithm/variable number was selected according to median performance across internal and external validations (5-fold cross-validation and 5-fold validation in Juin, Buffa, and Guedj cohorts, respectively). For each retained algorithm model, candidate genes were selected when they were present among the 200 first ranked important variables in at least three of five cross-validation (except for SVM derived model due to lack of accessibility to important variables).

Candidates genes from the seven selected models were analyzed by enrichment analysis using ToppFun from ToppGene suite (20), then results were pooled (pathway number limit = 50 per model). For each ToppGene category, significant enriched pathways (FDR < 0.05) were classified in subcategories using keywords search. The keywords used to define ToppGene subcategories (namely nervous system, immune cells, CAF, blood circulatory system, ECM, adipocyte, cell cycle and/or proliferation, DNA damage and/or repair and breast cancer) are detailed in Supplementary Fig. S2 legend. ToppGene pathway names were shortened on the heatmap for viewing purpose. Genes from nervous system–related pathways from all categories were combined, and their main function and/or pathway was determined using Uniprot and Entrez Gene databases. Genes whom the main function and/or pathway was specifically linked to nervous system were retained for the first GES.

### Transcription Factor Analysis

All genes were ranked according to *t* test (*limma*) between patients with relapse versus patients with no relapse, and gene set enrichment analysis (GSEA, *fgsea*) was performed on ranked genes for transcription factor (TF) enrichment using “TFT_Legacy” from molecular signature database (*msigdbr*). Enriched TFs (*P*_adjusted_ < 0.1) were selected and further analyzed for their canonic role using gene ontology database (*clusterProfiler*). Significant enriched pathways (*P*_adjusted_ < 0.05) were classified into 16 subcategories.

### Second Signature Generation

Same ML analyses than above were performed using feature selection based on biological knowledge and followed by dimensionality reduction instead of *t*-test feature selection as a first step. For that, 418 nervous system pathways were selected in Molecular Signatures Database (MSigDB), and enrichment score for each pathway was calculated for each patient using gene set variation analysis (GSVA, *GSVA*). Response prediction models based on the 418 pathway scores were created using eight tuned algorithms, and the first 50 important pathways were retained for each. Pathways found in at least three of five cross-validations were pooled, and leading-edge genes were extracted using *fgsea* package based on gene *t*-test ranking. Genes for whom main function and/or pathway was specifically linked to nervous system were retained for the second GES.

For both nervous system-related signatures, GES score was calculated using weighted average expression (expression sum of downregulated genes (*t* test, *t* <0) subtracted to expression sum of upregulated genes (*t* test, *t* >0), divided by the total number of upregulated and downregulated genes).

### Survival and Regression Analysis

Kaplan–Meier survival analysis was performed for RFS or OS in patients split according to median GES score (*survival* and *survminer*). Cox proportional hazard models were calculated for RFS or OS in patients split according to median score for univariate analysis (*survival*). For early versus late relapse analysis, minimum threshold for relapse class has been set to 7 patients and at least 10% of the total population. Age, chemotherapy option, tumor size, lymph node status, and histologic grade were used in combination with signature score for multivariate analysis. Log-rank test was used to calculate *P* values and Efron approximation was used for tie handling.

Prognostic and predictive capacities of the GES were both tested in this study. Prognostic term refers to an effect associated with recurrence, whether the patient is treated or not, and predictive term refers to a variable whose presence influences response to a treatment. Because treatment aims to decrease relapse, predictive factors are also influencing the prognosis of patients.

### Published Signatures

Scores for various already published GES were calculated according to published equations. *Genefu* package was used for calculation of OncotypeDx, EndoPredict, Genomic grade index (GGI), PAM50ROR, Gene70, GeniusM3, TAMR, PIK3CAGS, and Gene76 scores. ExagenBC, Mammostrat, Celera, TwoGeneRatio and Toronto95 scores were calculated as explained in Sjöström and colleagues publication (21). Signature scores were calculated only if at least 70% genes were available on microarray. Average signature was based on the scaled score (*z*-score) mean of the 16 signatures (14 published and 2 custom nervous-related signatures).

### CIBERSORTx, xCell, and Neurogenesis/Axonogenesis/Perineural Invasion Signatures

Nervous cell type abundance in bulk transcriptomics data was estimated by CIBERSORTx (22) using three different datasets as cell type reference. Neurogenesis was estimated by quantification of neural progenitor cells (NPC) at various stages of differentiation from stem cells to neurons using Wang and colleagues study (ref. 23; single-cell RNA-seq, GSE102066) and GSE65369 dataset (bulk-sorted microarray). Neural precursors from human cortex at three developmental stages were estimated using Johnson and colleagues study (ref. 24; GSE66217, bulk RNA-seq after FACS). All CIBERSORTx experiments were performed in absolute mode, with batch normalization and 100 permutations. Mesenchymal stem cell, multipotent progenitor, astrocyte, and neuron abundances were estimated using xCell (25).

Perineural invasion (PNI) was estimated using four scores derived from weighted average expression from GES extracted from four studies (26–29). Angiogenesis was estimated from weighted average expression using two scores extract from “Hu_Angiogenesis geneset” from MSigDB and Massiero and colleagues study (30). MSigDB “Hallmark_Angiogenesis geneset” score was also estimated using GSVA (*GSVA*) to compare results with the ones obtained in ref. 9. Axonogenesis was estimated using single-sample GSEA (ssGSEA, *GSVA*) derived score from a 70-gene list from Yang and colleagues study (31).

### SPADE Clustering

Spanning-tree progression analysis of density-normalized events (SPADE) clustering was performed with *spade* package on *in silico* sorting analysis of 64 immune and stromal cell types determined by xCell. Sorting scores were first transformed to density and stored in “FCS” file using *flowCore,* then clustering was performed using 50 nodes to generate unified trees based on the estimation of the 64 cell type abundance. Brain neural progenitor abundance, PNI, axonogenesis, angiogenesis and neuronal GES were not used for clustering, but their density scores were manually annotated on NervSign97 density results.

### Data Availability

The data analyzed in this study were obtained from GEO, European Nucleotide Archive (ENA) and cBioportal at GSE42568, GSE7390, GSE26971, EMTAB365, GSE25055, GSE20685, GSE19615, GSE6532, GSE9195, GSE2603, GSE5327, GSE45255, GSE1456, GSE31519, GSE11121, GSE17907, GSE17705, GSE2034, GSE12093, GSE7378, GSE22219, GSE9893, GSE58812, GSE140489, brca_metabric and GSE96058.

## Results

### Patient Selection and Algorithm Pipeline Configuration

To select important genes linked to response to treatment in breast cancer, ML analysis was performed on patients with breast cancer for whom naïve tumor transcriptome and response to treatment were known (pipeline in Supplementary Fig. S1A). Among the choice of clinical efficacy endpoints (pathological complete response after neoadjuvant treatment, relapse and survival), relapse was selected as the most relevant outcome for treatment resistance biomarkers. To gather sufficient statistical power, we combined datasets available in public databases using ComBat cross-platform normalization (Supplementary Fig. S1B). In that way, 3,940 patients from 21 studies were gathered. Patient characteristics are presented in Supplementary Table S2.

From this step, we focused on patients treated by hormonotherapy with known relapse status. Among the 1,480 selected patients, 350 had relapse and 1,130 patients were relapse free. Because imbalanced class population causes bias toward the majority class in ML analysis, six resampling methods were tested to counteract this effect (Supplementary Fig. S1C).

**FIGURE 1:**
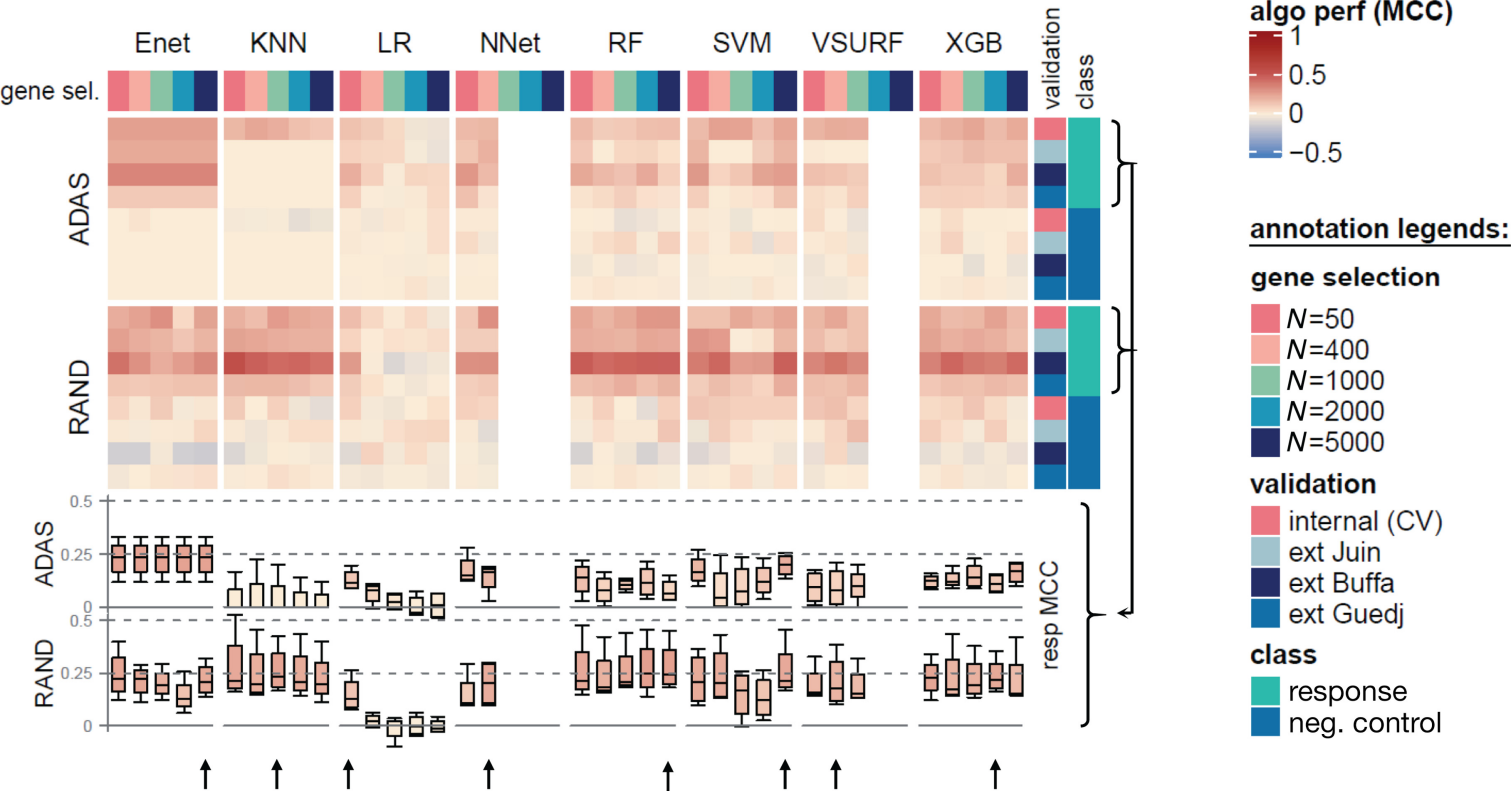
Model development for response to treatment prediction. Performance for eight classifier algorithms for best undersampling (random) and oversampling (ADASYN) methods were estimated on internal (cross-validation) and external (ext) validation (Juin, Buffa, and Guedj cohorts) using with five variable selection options. Class to predict was either treatment response endpoint, or negative control (randomly assigned class). Performance was calculated using MCC. Box plot for treatment response endpoint combining internal and external validation were plotted in bottom annotation. Arrows indicates the selected model in random undersampled dataset.

We then developed 70 models for response to treatment prediction by applying different options including two balanced datasets (ADASYN oversampling and random undersampling), eight predictive algorithms, and five choices of feature selection number (to minimize feature of the high-dimensional dataset). Prediction performances were tested by cross-validation (internal validation) and on three external cohorts. The same analysis was performed with a randomly picked class as negative control (Fig. 1). The best models had median internal validation MCC between 0.15 and 0.29 according to algorithm. These results were within the same range than the ones obtained by MAQC2 consortium for event-free survival as endpoint (0.16 and 0.42 for multiple myeloma and neuroblastoma, respectively; ref. 32). We noticed that external validation prediction performance was in the same range or better than the internal cohort prediction for the undersampled dataset (counting 568 patients), suggesting that the models were not overfitted. On the contrary, oversampling increased overfitting bias. We selected the best performer for each algorithm in undersampled dataset (see arrows in Fig. 1), and extracted the important genes linked to relapse after hormonotherapy (available for all but SVM model).

### Neural System is a Marker of Resistance to Hormonotherapy in Patients with Breast Cancer

**FIGURE 2:**
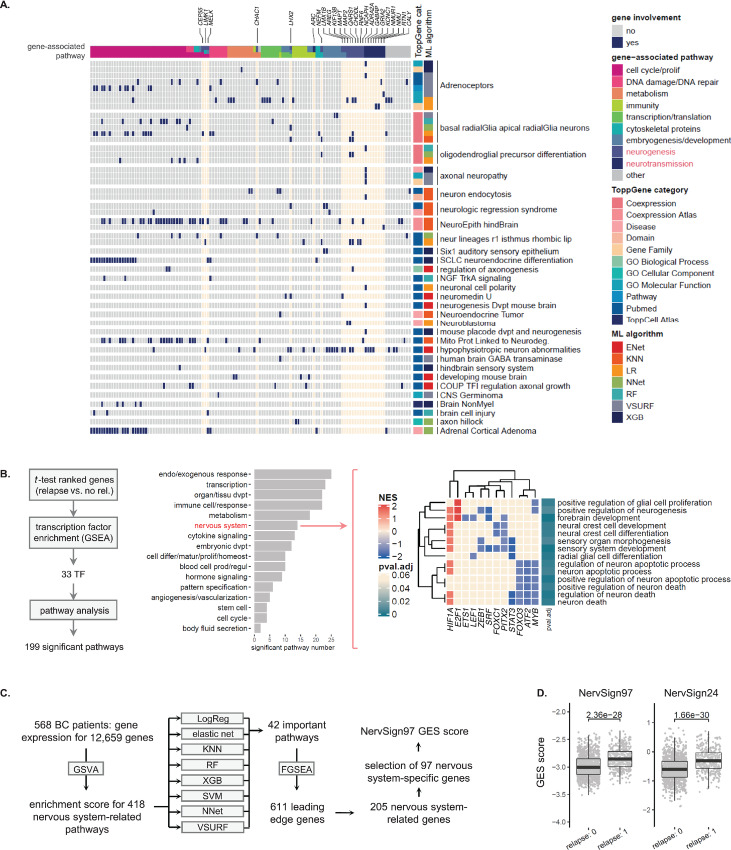
Neural System is a marker of resistance to hormonotherapy in patients with breast cancer. **A,** A ToppGene analysis was performed on union of the 100 first important genes derived from seven ML models for relapse prediction in the 568-patient cohort, and statistically relevant associated pathways were listed for each ToppGene categories (see Supplementary Fig. S2A and S2B). Genes from pathways related to nervous system were plotted on heatmap according to their pathway belonging, and annotated with their main function. The 24 genes specifically associated with neurogenesis and neurotransmission were indicated at the top and will be used as first signature (the whole gene list is presented in Supplementary Fig. S2C). **B,** TF analysis was performed on patients according to response to treatment, and gene ontology (GO) pathway analysis was performed on significantly “activated” TFs. TF-related pathways were plotted according to subcategories (middle) and the nervous system subcategory was detailed on a heatmap on the right panel. **C,** The second nervous system related signature (NervSign) was obtained following a pipeline using genes related to nervous pathway only (see details in Materials and Methods). **D,** Box plot of Nervsign24 and NervSign97 weighted average expression scores according to relapse in hormonotherapy-treated patients (unbalanced merged Affymetrix cohort, *n* = 1,480). Statistical significance between groups was assessed using unpaired two-tailed Student *t* test.

To build a signature linked to TME neuronal components only, we filtered the ML-selected biomarkers according to biological knowledge. First, functional enrichment analysis of the seven gene lists was used to determine the involvement of selected pathways and TME components in response to treatment. It revealed—as expected—that cell-cycle process/proliferation was the main pathway associated with hormonotherapy response (Supplementary Fig. S2A and S2B). When looking at the TME components (immune cells, CAFs, blood circulatory system, ECM, adipocytes and nervous system), we observed that the nervous system was the second most important component after immune cells (Supplementary Fig. S2B, bottom). At the gene level, on the 341 genes issued from ML analysis, 124 were involved in nervous system-related pathways. To further refine the nervous system-related biomarkers, the main associated functions in each of the 124 neuronal-related genes were investigated and reported on a heatmap on Fig. 2A (see Supplementary Fig. S2C and Supplementary Fig. S3A for the generated gene lists). Among them, more than a third was mainly associated with cell cycle and proliferation, and only 24 genes were specifically associated with neural function (neurogenesis and neurotransmission), with 12 downregulated genes and 12 upregulated genes. These 24 genes were retained as a first nervous system–related GES.

To confirm functional enrichment results, we performed a TF enrichment analysis that highlighted 33 activated TF, defined according to their upregulated and downregulated targets (Fig. 2B). When looking more precisely at the enrolled TF pathways, the nervous system appeared in sixth position (second position for TME components), with neurogenesis/neural development and neuron apoptosis as principal associated mechanisms.

Having confirmed the presence of nervous system biomarkers in features linked to relapse, we derived a second ML model by testing another feature reduction method based on biological filtering (instead of *t*-test filtering), followed by dimensional reduction. The new ML analysis was performed on nervous system-related pathways uniquely (see the pipeline in Fig. 2C and the generated gene lists in Supplementary Fig. S3B). A total of 97 genes were selected as nervous system-specific genes associated with response to hormonotherapy: 66 genes were downregulated and 31 were upregulated (see Supplementary Fig. S4A). The 97-gene signature shared seven genes with the above-cited 24-gene signature.

Two GES named NervSign97 and NervSign24 were derived from the two gene lists. We then compared the performance of the GES to select the more robust one. Gene expression score for each was calculated by weighted average expression (see details in Methods). As observed in Fig. 2D, the two nervous signature scores in the merged Affymetrix hormonotherapy-treated cohort (*n* = 1,480, including 568 patients from the ML analysis subcohort) were strongly associated with relapse (*P* = 2.36e-26 and 1.37e-30). Other prognostic variables (tumor size, axillary lymph node status, histologic grade and age) were also associated with both signature scores (see Supplementary Fig. S4B). Because of similar performances of the two GES in this first validation assay, we continued to compare their performances in the next validation steps.

### Validation of the Two Nervous System–related Signatures

**FIGURE 3:**
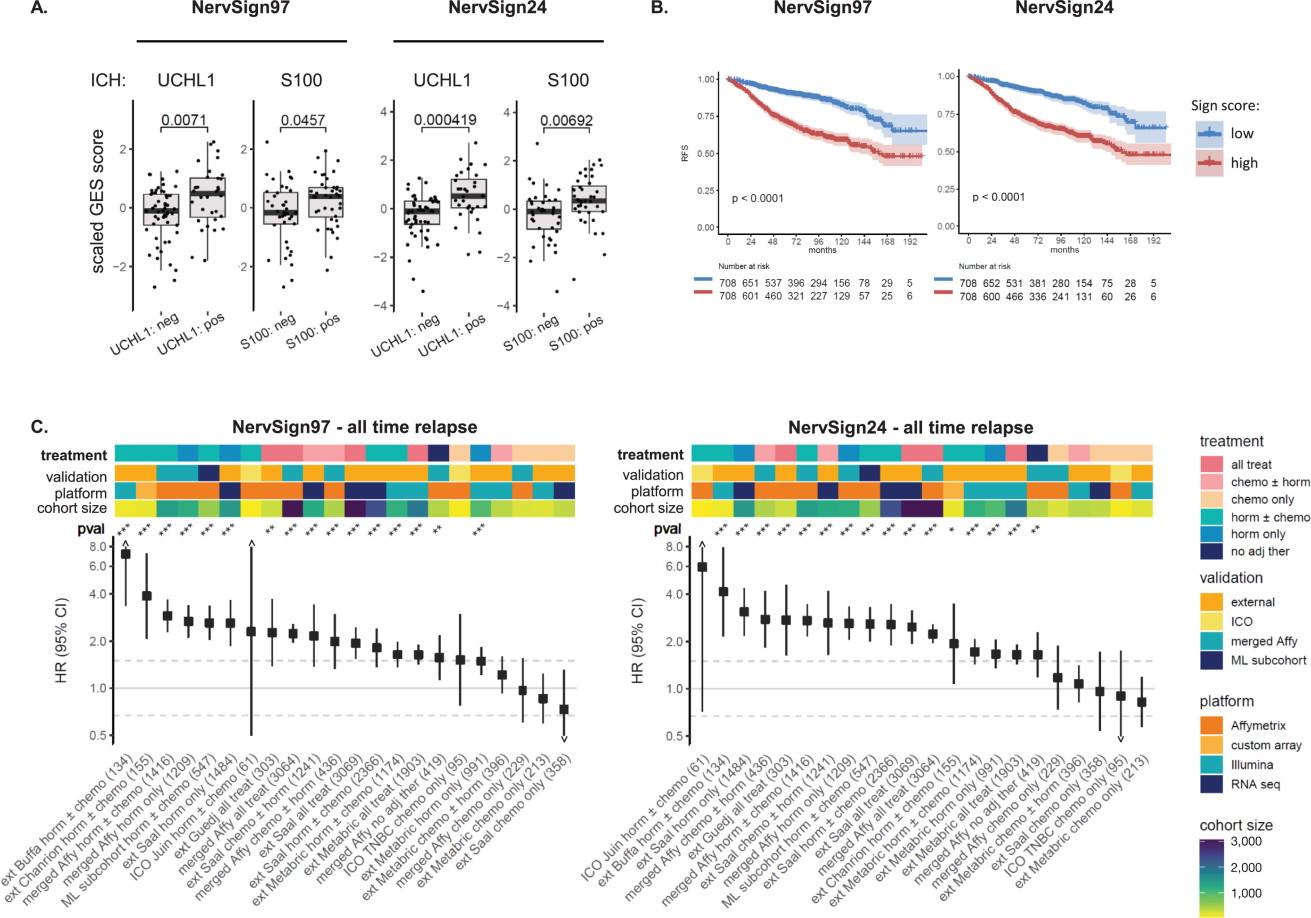
Validation of the two nervous signatures in internal and external cohorts. **A,** Association of NervSign scores with IHC nervous markers status (UCHL1 and S100) in a 107-patient cohort (*n* = 85 and *n* = 80, respectively). Statistical significance between groups was assessed using unpaired two-tailed Student *t* test. **B,** RFS Kaplan–Meier analysis was performed in patient cohort stratified according to nervous signature median score (hormonotherapy-treated patients, unbalanced merge Affymetrix cohort, *n* = 1,416). The shaded area represents the 95% CIs. **C,** HRs with 95% CI and *P* values (pval) were calculated using Univariate Cox analysis in several cohorts stratified according to NervSign97 or NervSign24 median score (***, **, and * for *P* value <0.001, 0.01, and 0.5, respectively). All HR were related to RFS except for Saal cohort (OS). “horm ± chemo” and “chemo ± horm” refer to patients treated with hormonotherapy or chemotherapy with no exclusivity, whereas “horm only” and “chemo only” indicate exclusivity. “External” refers to external validation cohorts, “ICO” to our hospital validation cohort, “merged Affy” to the initial merged Affymetrix cohort, and “ML subcohort” to the subset population used to create ML-derived gene signatures. Numbers between parentheses indicate cohort size.

Association of the two GES with IHC neural markers (S100 and UCHL1) was analyzed in a 107-patient cohort from ICO (19). For both, increased scores were significantly linked with positive protein marker statuses (Fig. 3A). Knowing that each S100 family protein is encoded by a separate gene, we have additionally ensured that S100B gene expression, downregulated in NervSign97, was not linked to S100 protein IHC status (see Supplementary Fig. S5A).

**FIGURE 4:**
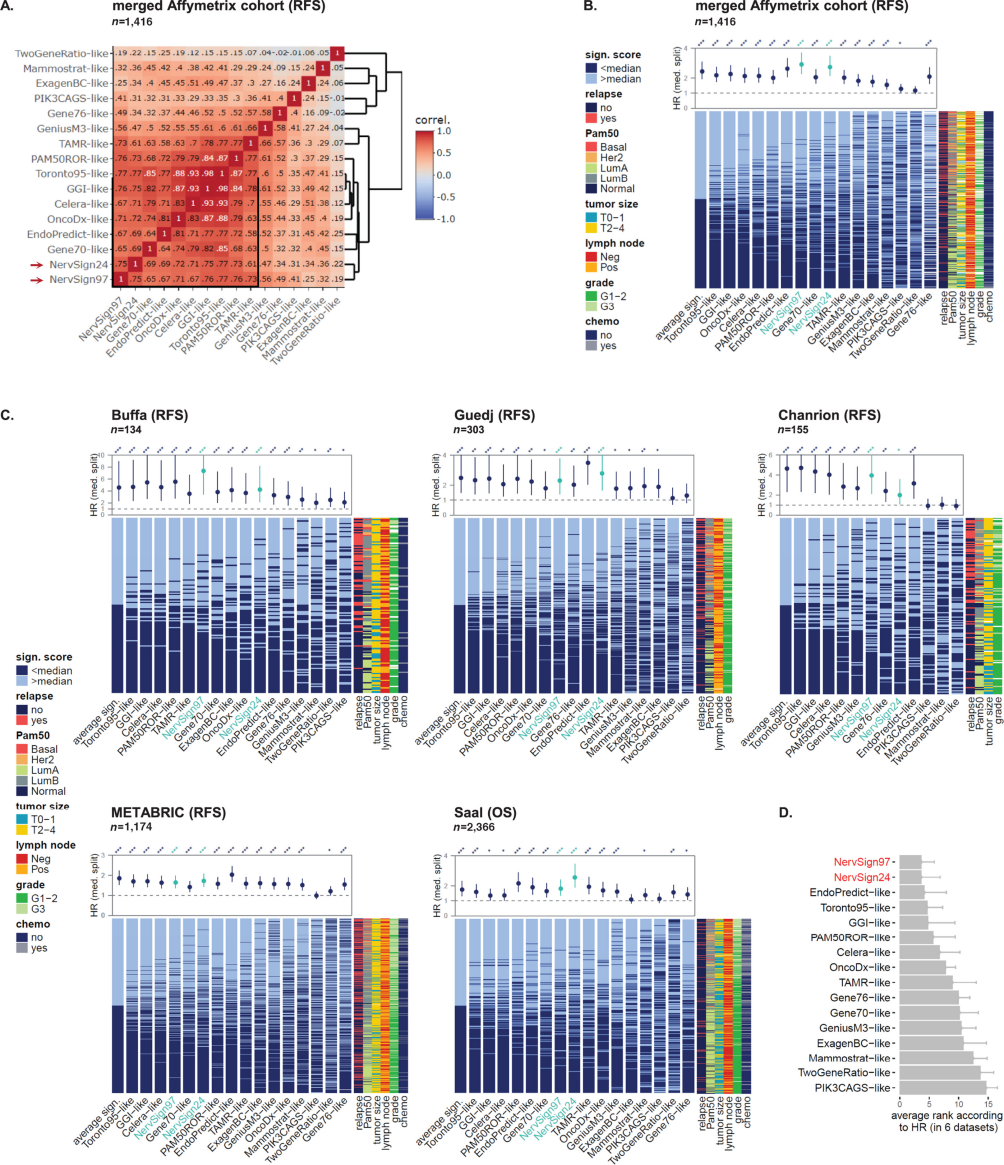
Comparison of previously published gene expression signatures in merged Affymetrix and independent cohorts. **A,** Pearson correlations between the 16 signatures scores were hierarchically clustered and plotted accordingly. The most related signatures were identified using the dendrogram and highlighted with a black square box. **B,** Comparison of NervSign24 and NervSign97 scores with 14 previously published signatures in hormonotherapy-treated cohort (*n* = 1,416). Signature scores were calculated for each patient (in row) and column-ordered according to average score (except Gene76-like when “NA” where generated because of ER status missing information). Bar plot was colored according to score median. Molecular type (PAM50), relapse, tumor size, lymph node, histological grade, and chemotherapy are presented in left annotation. For each signature score, HRs from Cox proportional hazards model were plotted at the top (***, **, and * for *P* value <0.001, 0.01, and 0.5, respectively), with 95% CIs. **C,** Same analysis than in **A** was performed in four external cohorts with either OS or RFS as endpoint, and either RNA-seq (Saal) or microarray (Illumina platform for METABRIC and Buffa, Affymetrix for Guedj) used for RNA quantification. All patients were treated with hormonotherapy except for Guedj cohort where 88% patients received hormonotherapy. **D,** Signatures are sorted according to their average rank score obtained using HR value in six cohorts (from **B** and **C**).

We investigated whether the two GES were predictive of recurrence, using univariate Cox models based on patients treated by hormonotherapy with known relapse and RFS statuses (*n* = 1,416, see cohort description in Supplementary Fig. S5B). As seen in Kaplan–Meier curves (Fig. 3B), high scores for both GES were significantly associated with unfavorable RFS in the merged Affymetrix hormonotherapy-treated cohort (*n* = 1,416, including 547 patient from the ML subcohort), with HR = 2.72 (95% CI = 2.15–3.43) for NervSign24 (*P* = 5.36e-19) and HR = 2.9 (95% CI = 2.29e-3.66) for NervSign97 (*P* = 3.4e-17). The same analysis performed with other prognostic variables (tumor size, lymph node, histologic grade) showed lower HRs with less significant *P* values compared with the results obtained with the two GES, and patient age was not significantly associated with RFS (Supplementary Fig. S5C).

We then assessed the specificity of the two GES by evaluating their performance according to adjuvant treatment (hormonotherapy or chemotherapy) for predictive abilities, or combining all patients (with and without adjuvant therapy) for prognostic abilities. This analysis was performed on the Affymetrix merged cohort (*n* = 3,084 patients with known RFS, including 547 patients from the ML subcohort), as well as in seven independent cohorts from various RNA quantification platforms and various size: Juin (Affymetrix, *N* = 61), Buffa (Illumina, *N* = 134), Guedj (Affymetrix, *N* = 303), Chanrion (custom array, *N* = 155), ICO TNBC (Affymetrix, *N* = 107), METABRIC (Illumina, *N* = 1,903), and Saal (RNA-seq/OS, *N* = 3,069). All results were compiled in a forest plot showing HR with 95% CI and *P*-value significance. Prognostic and predictive capacities of the two signatures were ranked according to HR score in the eight cohorts (with treatment specificity indicated above forest plot). Variables known to generate biases (cohort batch, RNA quantification platform, and cohort size) were also indicated above plot, to check whether GES performances were influenced with any of them (Fig. 3C). For both GES, patients with high signature score were significantly associated with worst outcome (HR > 1.5 and *P* < 0.001) in almost all configurations except for chemotherapy-only treated subgroups. This indicates that NervSign97 and NervSign24 are prognostic and hormonotherapy-predictive signatures, but not chemotherapy-predictive. Both GES were robust enough not to be susceptible to platform bias or cohort batch. Statistical significance was only affected in the smallest cohort (Juin, *n* = 61 including seven patients with relapse) for whom the wide 95% CI indicated unexploitable results. This cohort was therefore not used in the signature performance comparison performed in the next result section.

Finally, we investigated whether the nervous system related signatures were linked to timing of recurrence by performing the same experiment in patients with either early or late recurrence (before/after 5 years). When looking at early recurrence only, results were similar to the ones obtained with all relapses combined (Supplementary Fig. S6A). Nevertheless, in late recurrence setting, results obtained in the validation cohorts (and particularly in METABRIC cohort) were not satisfying enough to conclude the same (Supplementary Fig. S6B).

### Comparison of the Two Signatures with Already Published Ones

To evaluate further the hormonotherapy-predictive ability of the two nervous-related GES, their performances were compared in six cohorts with 14 already published GES, including four hormonotherapy-predictive GES (TAMR, PI3CAGS, Toronto95, TwoGeneRatio), two chemotherapy-predictive GES (OncotypeDX, EndoPredict) and eight prognostic GES (PAM50ROR/Prosigna, Gene70/Mammaprint, Gene76/Rotterdam, GGI, Celera, Genius3M, ExagenBC, Mammostrat). Only hormonotherapy-treated patients were kept in this analysis. Only 13 signatures scores were calculated in the Chanrion cohort, due to the non-availability of some genes in the custom microarray. In all tested cohorts except the Chanrion's (merged Affymetrix, Buffa, Guedj, Saal and METABRIC), most of GES risk scores were highly correlated to each other. Toronto95, GGI, and Celera were the highest correlated (above 0.9), and TwoGeneRatio, PIK3CAGS and Mammostrat were the lowest, with absolute correlation coefficients below 0.5 (Fig. 4A; Supplementary Fig. S7). NervSign24 and NervSign97 were always found in the highly correlated cluster (black-square box). Correlation coefficients with the other same-cluster GES were between 0.55 and 0.83 for NervSign24, and between 0.63 and 0.79 for NervSign97 (not considering Chanrion cohort). To investigate further disparity/resemblance between GES, we sorted them according to correlation to average GES (mean of the 16 GES scores). Despite some disparities between individual patients, median splits were quite similar across signatures for the first half of the GES correlated to average (Fig. 4B and C). We therefore performed univariate Cox proportional hazard models for all GES in the six cohorts by stratifying patients according to median score, and plotted HRs with 95% CI above the corresponding signature score results (Fig. 4B and C). Among the 16 GES, six were predictive in the six tested cohorts, with HR > 1.5 and associated *P* value <0.05: NervSign97, NervSign24, EndoPredict, Toronto95, PAM50ROR, and GeniusM3. To further compare the performances of the 16 GES, we ranked them according to HR value in each cohort, and calculated the resulting average rank for each. NervSign97 and NervSign24 were ranked first and second (Fig. 4D), emphasizing their strong capacity to stratify patients with increased risk of relapse.

To adjust for confounders, a multivariable Cox analysis was done in the largest cohorts (merged Affymetrix, METABRIC and Saal) with NerSign24 or NervSign97 in combination with available clinically relevant covariates (age, cotreatment with chemotherapy, tumor size, lymph node status, and histologic grade). Both GES retained their association with RFS in the three cohorts, as well as tumor size (see multivariate analyses in Supplementary Fig. S8A vs. univariate analyses in Supplementary Figs. S5C and S8B). For the other covariates, all remained significant in Saal cohort, whereas only lymph node status remained significant In METABRIC cohort (for both GES as covariate) and in Affymetrix cohort (with NervSign24 as covariate).

From this step, we focused on NervSign97 because it was ranked first in the HR-based average ranking across cohorts, and it had associated *P* value <0.001 in all tested cohort (whereas NervSign24 had *P* value <0.001 in five cohorts and *P* value <0.05 in one cohort).

### NervSign97 is Associated with a Type of Brain Neural Precursors and Perineural Invasion

**FIGURE 5:**
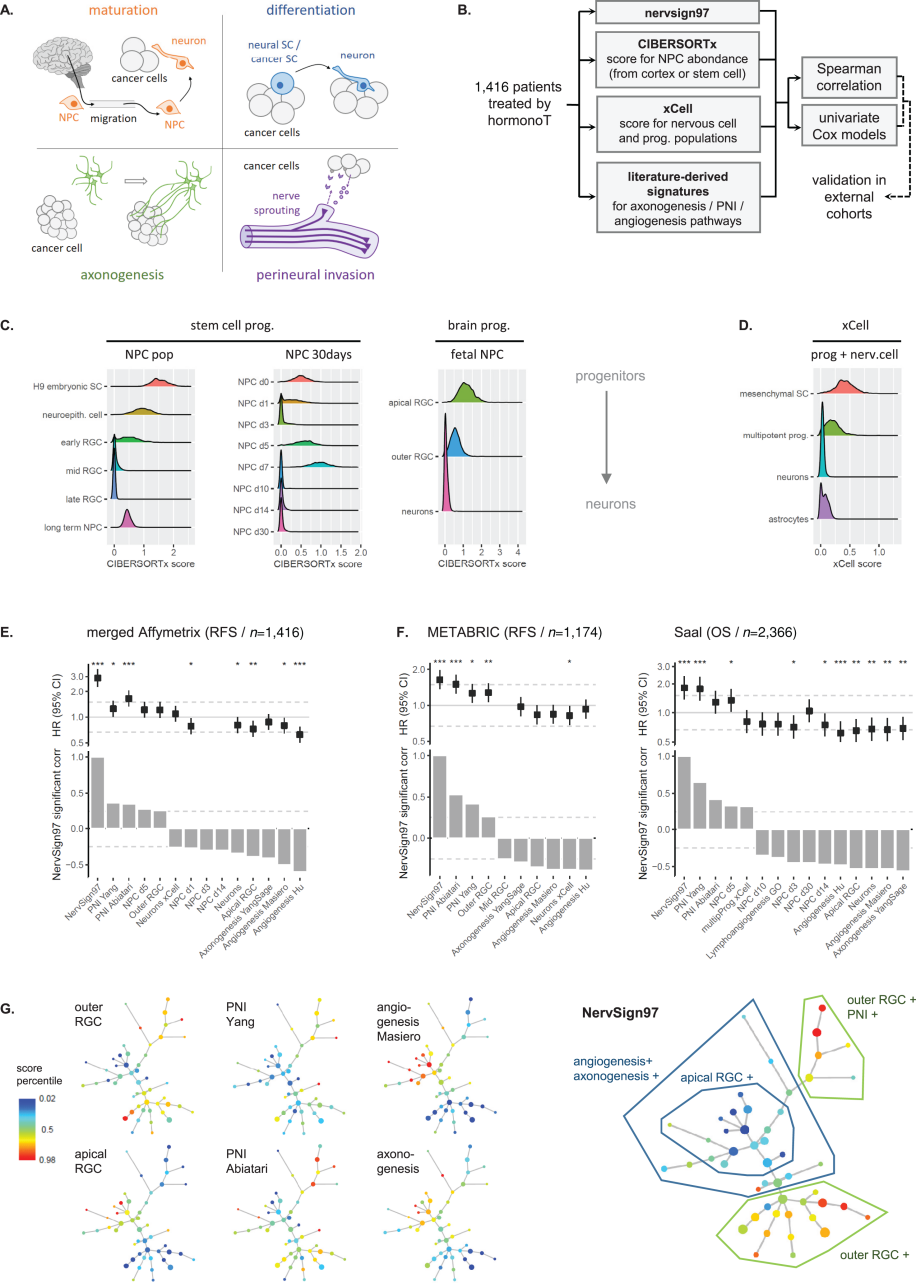
NervSign97 is associated with brain neural precursors and perineural invasion. **A,** Schematic representation of the four contributions of the nervous system in a tumor (NPC: neural progenitor cell, SC: stem cell). **B,** Pipeline of investigation for NervSign97-associated nervous system contribution. **C,** NPC presence across samples were plotted from data generated by CIBERSORTx (RGC = radial glial cells). It assigns a score of arbitrary units that reflects the absolute proportion of each cell type in a mixture. **D,** Same analysis than in **C**. was performed using progenitor and neural cell estimation from xCell analysis (SC: stem cell, prog: progenitor). Significant Spearman correlations between NervSign97 score and nervous system contribution scores (stem cell progenitor, brain progenitor, PNI, and axonogenesis/angiogenesis) were calculated in merged Affymetrix cohort (**E**) and METABRIC and Saal cohorts (**F**). Cox model hazard ratios for significantly correlated contributions were plotted at the top (***, **, and * for *P* value <0.001, 0.01, and 0.5, respectively) with 95% CIs. **G,** SPADE clustering was performed in 1,416-patient cohort according 64 immune and stromal (including neural) cell type abundance estimation by xCell. Node size is proportional to the number of patients showing similar scoring. Each tree is colored according to either brain NPC abundance estimation, selected pathway signature or NervSign97 scores (that were not used for SPADE clusterization).

We then analyzed more precisely the underlying neuronal mechanism associated with NervSign97. Indeed, according to literature (reviewed in ref. 6), there are four mechanisms describing the emergence of neural cells in TME (see graphic representation in Fig. 5A). The first one involves NPCs from brain cortex that migrate toward tumor to maturate into neuronal cells once infiltrated in tumor. The second is local cancer stem cells that differentiate into NPCs first, then to neural cells. The third mechanism is axonogenesis, where nervous cells adjacent to the tumor develop axons toward tumor for its innervation. The fourth is PNI, where tumor cells invade locally existent nerves with a costimulation of growth. The two first mechanisms involve neurogenesis while the two last are interactive processes between tumor and already existing neural cells.

We developed a pipeline to decipher which of these four mechanisms was associated to NervSign97 (Fig. 5B). Axonogenesis and PNI were assessed using previously published GES. We also evaluated angiogenesis because it is often described to occur concomitantly with axonogenesis. To identify the two neurogenesis-related mechanisms, we used CIBERSORTx and xCell, two digital cytometry tools that estimate cell type abundance in bulk tissue (22, 25). CIBERSORTx was used to evaluate the proportion of cancer stem cell NPCs or brain NPCs in tumors based on three published NPC transcriptomes. xCell was used to determine mesenchymal stem cells, multipotent progenitors, astrocytes, and neurons proportions in tumor. CIBERSORTx analysis on the Affymetrix hormonotherapy-treated cohort (*n* = 1,416) indicated the presence of various NPCs (cancer stem cell-derived and brain-derived) in patient tumors, and a small proportion of mature neurons (Fig. 5C). xCell analysis confirmed that differentiated neurons was not an abundant population in tumors whereas progenitor cells were (Fig. 5D).

We then performed a correlation analysis in the 1,416-patient cohort between NervSign97 score versus neural cell abundance, PNI, axonogenesis, and angiogenesis (Fig. 5E; Supplementary Fig. S9). Two PNI signatures were positively and significantly associated with NervSign97 (0.37 and 0.35), as well as the brain neural progenitors named outer radial glial cells (outer RGC, 0.25). On the contrary, axonogenesis and the two angiogenesis GES were negatively correlated with NervSign97 (−0.41, −0.5, and −0.6, respectively), as well as apical RGC (early brain progenitors, −0.39) and neurons (−0.34). The relevance of the NervSign97-correlated neural mechanisms in treatment response was also assessed by univariate Cox analysis. The two positively correlated PNI signatures were significantly associated with poor prognosis, while angiogenesis, neurons, and apical RGC (all inversely correlated with NervSign97) were significantly associated with a better clinical outcome. The same analysis was performed in METABRIC and Saal cohorts (selecting hormonotherapy-treated patients only), and confirmed the results (Fig. 5F).

Finally, we investigated whether NervSignature97 was associated with a uniform mechanism systematically including both increased PNI and increased outer RGC abundance, or whether there were subsets among patients. For that, we used an unsupervised algorithm (SPADE (33)), that organizes cells into hierarchies of related phenotypes. Clustered trees were constructed by applying SPADE to abundance estimation of 64 immune and stromal cells known to compose microenvironment (xCell). Three subsets were defined in that way (Fig. 5G) and colored according to the neural pathways or brain-derived NPCs that were significantly correlated with nervSign97 in Fig. 5E and F. Two subsets showed high nervSign97 scoring: the first one was outer RGC-positive, and the second was PNI plus outer RGC-positive. The subset with NervSign97 low scoring showed increased axonogenesis/angiogenesis or increased apical RGC/axonogenesis/angiogenesis.

In conclusion, NervSign97 signature allowed defining two subgroups of patients treated with hormonotherapy with higher risk of relapse: the first one combining PNI and brain neural progenitor signaling, and second one with mainly brain neural progenitor signaling.

## Discussion

In breast cancer therapy, one of the main goals is the improvement of treatment individualization through molecular target profiling (3). Not only biomarkers help stratifying patients to optimize selection of patients receiving a treatment, but they also open doors to new targeted treatments when the newly discovered biomarkers help to reveal a new underlying biological mechanism. Here, we developed a method focusing on the findings of new biological targets in TME, and we derived two GES related to TME neuronal components. The two neuronal GES were prognostic and hormonotherapy-predictive; they performed well in various validation cohorts, and were the best performers compared the published ones for hormonotherapy prediction.

Solid tumors are known to build a microenvironment to maximize their development, but neural component role in tumor progression has been longtime overlooked. New insights on the involvement of the nervous system on tumor has revealed new and diverse mechanisms showing similarities with embryonic development, tissue repair, and regeneration (14), and have drawn a strong interest among researchers to study the mechanisms behind it more thoroughly. PNI was a longtime identified mechanism, and initially thought to be a potential roadway for cancer cell dissemination, but more recent evidences indicated that it is a niche in perineural space where both nerves and cancer cells secrete local factors that are beneficial for each other growth (34). PNI is also a marker of aggressive tumor phenotype and poor prognosis in breast, pancreatic, gastric, prostate, colorectal, head and neck, and bile duct cancers (34). The newly discovered neuronal-related mechanisms (axonogenesis, cancer stem cell neural progenitor, and brain neural progenitor) are more difficult to expose clinically due to lack of appropriate markers and the fact that these phenomena are often entangled. For example, several studies reported that neurogenesis was linked with poor outcome (35, 36), but it was assessed by nerve density measurement using IHC S100 or UCHL1, which cannot distinguish PNI from neurogenesis. Adrenergic nerve density was also associated with axonogenesis, neurogenesis and poor clinical outcome (37, 38), but assessed with IHC tyrosine hydroxylase marker that cannot discriminate the distinct phenomenon either. Central nervous system progenitor implication in cancer development was discovered for the first time in 2019 in prostate cancer (39). In this study, brain NPCs were associated with poor clinical outcome in a 32-patient cohort using the immunofluorescent *DCX* marker, a protein involved in neuronal migration and neurogenesis. Outer radial glia-like cancer stem cells were also associated with tumor invasion in glioblastoma through *PTPRZ1* (40).

When looking specifically at the relationship between the nervous system and breast cancer, several studies have already reported interaction. Nerve fiber infiltration has been linked to pain, lymph node invasion or aggressive tumor progression (41–43). Another study showed that sympathetic nerves are linked with tumor progression, while parasympathetic innervation of tumors decelerates tumor growth, and these phenomena were associated with recurrence and decreased recurrence, respectively, in a 29-patient cohort (13). Two teams, including ours, reported that neurogenesis-related gene expressions were upregulated in a subgroup of TNBC, but it was not associated with clinical response in either studies (19, 44). PNI has also been investigated for its relationship with treatment outcome, but results were not always concordant: PNI has recently been associated with locoregional recurrence (12), but a former study reported a lack of association with relapse (11).

Because angiogenesis and neurogenesis/axonogenesis are linked (6), we analyzed the correlation between angiogenesis and NervSign97. Our results showed that increased angiogenesis was linked to lower NervSign97 GES score and better prognosis, but another study using GES demonstrated that increased angiogenesis was linked to poor prognosis (9). It is not the first time that angiogenesis gave discordant results related to breast cancer outcome (reviewed in ref. 45). GES scoring method is probably the reason here. Oshi and colleagues (9) used “hallmark angiogenesis” from MSigDB with GSVA scoring method. It calculates global gene enrichment without considering gene overexpression or underexpression. We used weighted average expression scoring, which includes gene expression direction. When we assessed the GES scoring method they used in three cohorts (merged Affymetrix, METABRIC, and Saal), we did not see any significant association between hallmark angiogenesis scoring and clinical outcome (see Supplementary Fig. S10A), contrary to what we observed with our scorings (Fig. 5E and F).

Overall, all these results advocate for the development of new biomarkers for the nervous system component detection. Even if IHC nervous markers exist, they have limited sensibility and are not indicative of the specific underlying phenomena. Accordingly, Guo and colleagues developed their own transcriptomic-based predictors to improve PNI detection (46). The development of more appropriated markers could help differentiate the specific phenomenon linked with cancer response, and appropriately define which one is involved with cancer prognosis.

We will also have to shed some light on the relationship between hormonotherapy and TME neuronal components in the future. The fact that the nervous system has been involved in clinical outcome in other cancers suggests a general phenomenon. Nevertheless, the neuronal GES that were developed in this study were hormonotherapy-predictive, but not chemotherapy-predictive. Several leads suggest a link between hormonotherapy and the nervous system. Estrogen is known to promote development, maturation, and function of the central nervous system (47). During early postnatal development, dorsal root ganglion neurons express ERα and ERβ, and the effect of 17β-estradiol on neuron survival *in vitro* is inhibited by antiestrogens including tamoxifen (47). In zebrafish, estradiol negatively regulates not only the proliferation, but also the migration of the newly generated RGC (48). In rats, estradiol treatment was associated with marks of increased radial glial migration (49).

We report here for the first time the possible involvement of outer RGC (also called basal RGC) in breast cancer outcome. Outer RGC are brain neural progenitors involved in neurogenesis. They are derived and closely related to apical RGCs (50). As described above, radial glial (RG) progenitors have already been involved in prostate and brain cancer poor outcome, and their association with estrogen was highlighted in zebrafishes and rats. In three breast cancer cohorts, we evaluated the link between hormonotherapy response and gene expression of the two previously used brain NPC markers (*DCX* and *PTPRZ1*). *DCX* was not related to RFS, and PTPRZ1 was correlated with good outcome for RFS (in METABRIC and merged Affymetrix cohorts, see Supplementary Fig. S10B). These data on brain progenitors are preliminary here, and would need further investigation to explore more in depth the relationship between brain neural progenitors, cancer development and hormonotherapy in breast cancer.

In conclusion, our study revealed the implication of neuronal cells in hormonotherapy response, thereby providing a new target worthy of further investigation in breast cancer therapy, as well as two robust GES to expose it clinically. It is nevertheless important to remember that this retrospective study was based on archived tumor specimens from literature where precise treatment information and detailed patient inclusion and exclusion criteria were not always available. Even if the signature robustness was proven by testing GES performance in several external validation cohorts and by a good ranking compared with the other published GES, NervSign24 and NervSign97 will have to be tested in a prognostic study to confirm their strength for future clinical use.

## Supplementary Material

Supplementary Data Supplementary Table S1: R package references
Supplementary Table S2: Merged Affymetrix cohort patient characteristics
Supplementary Table S3: Validation cohort patient characteristics
Supplementary Table S4: Algorithm parameter optimization
Supplementary Figure S1: Patient selection and algorithm pipeline configuration
Supplementary Figure S2: Generation of NervSign24
Supplementary Figure S3: ML-generated gene lists for the first signature and second signature generations
Supplementary Figure S4: Supplementary information related to Figure 2
Supplementary Figure S5: Supplementary information related to Figure 3
Supplementary Figure S6: Univariate Cox analyses in cohorts of patients with early or late recurrence
Supplementary Figure S7: Supplementary information related to Figure 4
Supplementary Figure S8: Multivariable and univariate Cox analyses in merged Affymetrix, METABRIC and Saal cohorts
Supplementary Figure S9: Correlation between nervous system mechanisms and NervSign97
Supplementary Figure S10: Kaplan-Meier analysis of previously published biomarkers
