# Supplementary material for: Brain Neural Progenitors are New Predictive Biomarkers for Breast Cancer Hormonotherapy
Source: Cancer Res Commun. 2022 Aug 24;2(8):857–69. doi: 10.1158/2767-9764.CRC-21-0090 (PMC10010318; doi:10.1158/2767-9764.CRC-21-0090)
Supplement: Supplementary Data — Supplementary Table S1: R package references Supplementary Table S2: Merged Affymetrix cohort patient characteristics Supplementary Table S3: Validation cohort patient characteristics Supplementary Table S4: Algorithm parameter optimization Supplementary Figure S1: Patient selection and algorithm pipeline configuration Supplementary Figure S2: Generation of NervSign24 Supplementary Figure S3: ML-generated gene lists for the first signature and second signature generations Supplementary Figure S4: Supplementary information related to Figure 2 Supplementary Figure S5: Supplementary information related to Figure 3 Supplementary Figure S6: Univariate Cox analyses in cohorts of patients with early or late recurrence Supplementary Figure S7: Supplementary information related to Figure 4 Supplementary Figure S8: Multivariable and univariate Cox analyses in merged Affymetrix, METABRIC and Saal cohorts Supplementary Figure S9: Correlation between nervous system mechanisms and NervSign97 Supplementary Figure S10: Kaplan-Meier analysis of previously published biomarkers [file crc-21-0090-s01.pdf]

packages	references
BEclear	Akulenko R, Merl M, Helms V. BEclear: Batch Effect Detection and Adjustment in DNA Methylation Data. PLoS One. 2016;11:e0159921.
caret	Kuhn M. Building Predictive Models in R Using the caret Package. Journal of Statistical Software. 2008;28:1–26.
complexeheatmap	Gu Z, Eils R, Schlesner M. Complex heatmaps reveal patterns and correlations in multidimensional genomic data. Bioinformatics. 2016;32:2847–9.
clusterProfiler	Wu T, Hu E, Xu S, Chen M, Guo P, Dai Z, et al. clusterProfiler 4.0: A universal enrichment tool for interpreting omics data. Innovation (N Y). 2021;2:100141.
FactoMineR	Lê S, Josse J, Husson F. FactoMineR: An R Package for Multivariate Analysis. Journal of Statistical Software. 2008;25:1–18.
fgsea	Korotkevich G, Sukhov V, Budin N, Shpak B, Artyomov MN, Sergushichev A. Fast gene set enrichment analysis [Internet]. 2021 Feb page 060012. Available from: https://www.biorxiv.org/content/10.1101/060012v3
flowcore	Hahne F, LeMeur N, Brinkman RR, Ellis B, Haaland P, Sarkar D, et al. flowCore: a Bioconductor package for high throughput flow cytometry. BMC Bioinformatics. 2009;10:106.
forestplot	Gordon M, Lumley T. forestplot: Advanced Forest Plot Using "grid" Graphics [Internet]. 2020. Available from: https://CRAN.R-project.org/package=forestplot
genefu	Gendoo DMA, Ratanasirigulchai N, Schröder MS, Paré L, Parker JS, Prat A, et al. Genefu: an R/Bioconductor package for computation of gene expression-based signatures in breast cancer. Bioinformatics. 2016;32:1097–9.
got	Wickham H. got2: Elegant Graphics for Data Analysis. [Internet]. Springer-Verlag New York; 2016. Available from: https://got2.tidyverse.org
glmnet	Friedman JH, Hastie T, Tibshirani R. Regularization Paths for Generalized Linear Models via Coordinate Descent. Journal of Statistical Software. 2010;33:1–22.
gPCA	Reese SE, Archer KJ, Therneau TM, Atkinson EJ, Vachon CM, de Andrade M, et al. A new statistic for identifying batch effects in high-throughput genomic data that uses guided principal component analysis. Bioinformatics. 2013;29:2877–83.
GSVA	Hänzelmann S, Castelo R, Guinney J. GSVA: gene set variation analysis for microarray and RNA-Seq data. BMC Bioinformatics. 2013;14:7.
kernlab	Karatzoglou A, Smola A, Hornik K, Zeileis A. kernlab - An S4 Package for Kernel Methods in R. Journal of Statistical Software. 2004;11:1–20.
limma	Ritchie ME, Phipson B, Wu D, Hu Y, Law CW, Shi W, et al. limma powers differential expression analyses for RNA-sequencing and microarray studies. Nucleic Acids Res. 2015;43:e47.
msigdbr	Dolgalev I. msigdbr: MSigDB Gene Sets for Multiple Organisms in a Tidy Data Format [Internet]. 2020. Available from: https://CRAN.R-project.org/package=msigdbr
nnet	Venables WN, Ripley BD. Modern Applied Statistics with S [Internet]. Fourth. New York: Springer; 2002. Available from: https://www.stats.ox.ac.uk/pub/MASS4/
preprocessCore	Bolstad B. preprocessCore: A collection of pre-processing functions [Internet]. 2020. Available from: https://github.com/bmbolstad/preprocessCore
randomForest	Liaw A, Wiener M. Classification and Regression by randomForest. R news. 2002;2:5.
rknn	Li S. rknn: Random KNN Classification and Regression [Internet]. 2015. Available from: https://CRAN.R-project.org/package=rknn
simpleaffy	Miller CJ. simpleaffy: Very simple high level analysis of Affymetrix data [Internet]. 2020. Available from: http://bioinformatics.picr.man.ac.uk/simpleaffy/
smotefamily	Wacharasak S. smotefamily: A Collection of Oversampling Techniques for Class Imbalance Problem Based on SMOTE [Internet]. 2019. Available from: https://CRAN.R-project.org/package=smotefamily
spade	Qiu P, Simonds EF, Bendall SC, Gibbs KD, Bruggner RV, Linderman MD, et al. Extracting a cellular hierarchy from high-dimensional cytometry data with SPADE. Nat Biotechnol. 2011;29:886–91.
survival	Therneau TM, Grambsch PM. Modeling Survival Data: Extending the Cox Model. New York: Springer; 2000.
survminer	Kassambara A, Kosinski M, Biecek P. survminer: Drawing Survival Curves using "got2" [Internet]. 2020. Available from: https://CRAN.R-project.org/package=survminer
sva	Leek JT, Johnson WE, Parker HS, Jaffe AE, Storey JD. The sva package for removing batch effects and other unwanted variation in high-throughput experiments. Bioinformatics. 2012;28:882–3.
VSURF	Genuer R, Poggi J-M, Tuleau-Malot C. VSURF: An R Package for Variable Selection Using Random Forests. The R Journal. R Foundation for Statistical Computing; 2015;7:19–33.
xgboost	Chen T, Guestrin C. XGBoost: A Scalable Tree Boosting System. Proceedings of the 22nd ACM SIGKDD International Conference on Knowledge Discovery and Data Mining. 2016;785–94.

Supplementary Table S1: R package references

batch + reference ID	platform (HG133)	relapse	follow-up median/end	time period	tumor size	lymph nodes	histological grade	ER	PR	HER2	hormono-therapy	chemo-therapy	median age [Q1-Q3]
Clarke (121)<br>GSE42568	2+ (121)	0 (56)<br>1 (48)<br>NA (17)	5.3 yrs<br>/ NA	1993-<br>1997	T1 (18)<br>T2 (78)<br>NA (17)	Neg (45)<br>Pos (59)<br>NA (17)	G1-2 (51)<br>G3 (53)<br>NA (17)	Neg (34)<br>Pos (67)<br>NA (20)	NA (121)	NA (121)	NA (121)	NA (121)	56 [49-68]
Desmedt07 (198)<br>GSE7390	A (198)	0 (107)<br>1 (91)	9.8<br>/ 10	1980-<br>1998	T1 (68)<br>T2 (129)<br>T3 (1)	Neg (198)	G1-2 (113)<br>G3 (83)<br>NA (2)	Neg (64)<br>Pos (134)	NA (198)	NA (198)	no (198)	no (198)	46 [42-51]
Filipits (277)<br>GSE26971	A (277)	0 (200)<br>1 (58)<br>NA (19)	5.8<br>/ 10	1990-<br>1995	T1 (106) / T2 (149)<br>T3 (9)<br>NA (13)	Neg (131)<br>Pos (100)<br>NA (46)	NA (277)	NA (277)	NA (277)	NA (277)	yes (277)	no (277)	NA
Guedj (528)<br>EMTAB365	2+ (528)	0 (402)<br>1 (116)<br>NA (10)	5.2<br>/ 15	NA	NA (528)	Neg (132)<br>Pos (297)<br>NA (99)	G1-2 (314)<br>G3 (194)<br>NA (20)	Neg (108)<br>Pos (398)<br>NA (22)	NA (528)	Neg (340)<br>Pos (51)<br>NA (137)	NA (528)	NA (528)	55 [47-64]
Hatzis (310)<br>GSE25055	A (310)	0 (244)<br>1 (66)	2.4<br>/ 3	2000-<br>2006	T0 (2) / T1 (20)<br>T2 (165) / T3 (74)<br>T4 (49)	Neg (87)<br>Pos (223)	G1-2 (136)<br>G3 (151)<br>NA (23)	Neg (131)<br>Pos (178)<br>NA (1)	Neg (162)<br>Pos (147)<br>NA (1)	Neg (291)<br>Pos (8)<br>NA (11)	no (131)<br>yes (178)<br>NA (1)	yes (309)<br>NA (1)	49 [43-58]
Kao (327)<br>GSE20685	2+ (327)	0 (244)<br>1 (83)	2.4<br>/ 3	1991-<br>2004	T1 (101) / T2 (188)<br>T3 (26) / T4 (12)	Neg (137)<br>Pos (190)	NA (327)	NA (327)	NA (327)	NA (327)	no (54)<br>NA (273)	no (54)<br>NA (273)	46 [40-54]
Li (115)<br>GSE19615	2+ (115)	0 (100)<br>1 (15)	NA<br>/ 3	NA	T1 (50)<br>T2 (58)<br>T3 (7)	Neg (62)<br>Pos (53)	G1-2 (51)<br>G3 (64)	Neg (45)	Neg (51)	Neg (79)	no (48)<br>yes (64)<br>NA (3)	no (31)<br>yes (81)<br>NA (3)	53 [45-60]
Loi07_1 (87)<br>GSE6532	2+ (87)	0 (59)<br>1 (28)	11.5<br>/ 10	1980-<br>1998	T1 (30)<br>T2 (53)<br>T3 (4)	Neg (29)<br>Pos (58)	G1-2 (54)<br>G3 (16)<br>NA (17)	Pos (87)	Neg (21)<br>Pos (64)<br>NA (2)	NA (87)	yes (87)	no (87)	62 [58-67]
Loi07_2 (327)<br>GSE6532	A+B (327)	0 (195)<br>1 (111)<br>NA (21)	6.1<br>/ 10	1980-<br>1998	T0 (6) / T1 (116)<br>T2 (179) / T3 (13)<br>NA (13)	Neg (221)<br>Pos (85)<br>NA (21)	G1-2 (210)<br>G3 (60)<br>NA (57)	Neg (45)<br>Pos (262)<br>NA (20)	Neg (11)<br>Pos (121)<br>NA (195)	NA (327)	yes (327)	no (327)	60 [51-68]
Loi08 (77)<br>GSE9195	2+ (77)	0 (64)<br>1 (13)	8.3<br>/ 10	NA	T1 (28)<br>T2 (46)<br>T3 (3)	Neg (41)<br>Pos (36)	G1-2 (34)<br>G3 (24)<br>NA (19)	Pos (77)	Neg (18)<br>Pos (59)	NA (77)	yes (77)	no (77)	65 [58-72]
Minn05 (121)<br>GSE2603	A (121)	0 (55)<br>1 (27)<br>NA (39)	5.4<br>/ 10	1990-<br>2001	T1 (11) / T2 (71)<br>T3 (17)<br>NA (22)	Neg (34)<br>Pos (65)<br>NA (22)	NA (121)	Neg (42)<br>Pos (57)<br>NA (22)	Neg (55)<br>Pos (43)<br>NA (23)	Neg (75)<br>Pos (13)<br>NA (33)	NA (121)	NA (121)	56 [46-64]
Minn07 (58)<br>GSE5327	A (58)	0 (47)<br>1 (11)	7.2<br>/ 10	1980-<br>1995	NA (58)	NA (58)	NA (58)	Neg (58)	NA (58)	NA (58)	NA (58)	NA (58)	NA
Nagalla (139)<br>GSE45255	A (139)	0 (104)<br>1 (32)<br>NA (3)	4.7<br>/ 10	1994-<br>2002	T1 (26)<br>T2 (102)<br>T3 (11)	Neg (94)<br>Pos (45)	G1-2 (69)<br>G3 (67)<br>NA (3)	Neg (48)<br>Pos (89)<br>NA (2)	Neg (60)<br>Pos (72)<br>NA (7)	Neg (69)<br>Pos (62)<br>NA (8)	no (53)<br>yes (78)<br>NA (8)	no (58)<br>yes (73)<br>NA (8)	53 [46-63]
Pawitan (159)<br>GSE1456	A+B (159)	0 (119)<br>1 (40)	7.1<br>/ 5	1994-<br>19A	NA (159)	NA (159)	NA (159)	Neg (25)<br>Pos (39)<br>NA (95)	NA (159)	NA (159)	NA (159)	NA (159)	NA
Rody (67)<br>GSE31519	A (67)	0 (39)<br>1 (23)<br>NA (5)	2.8<br>/ 5	19A-<br>2007	T1 (64)<br>NA (3)	Neg (44)<br>Pos (21)<br>NA (2)	G1-2 (18)<br>G3 (45)<br>NA (4)	Neg (67)	Neg (67)	Neg (67)	no (67)	no (9)<br>yes (58)	NA
Schmidt (200)<br>GSE11121	A (200)	0 (154)<br>1 (46)	7.5<br>/ 5	1988-<br>1998	T1 (99)<br>T2 (96)<br>T3 (5)	Neg (200)	G1-2 (165)<br>G3 (35)	NA (200)	NA (200)	NA (200)	no (200)	no (200)	NA
Sircoulomb (55)<br>GSE17907	2+ (55)	0 (22)<br>1 (17)<br>NA (16)	2.7<br>/ 5	1987-<br>2007	T1 (7) / T2 (15)<br>T3 (12)<br>NA (21)	Neg (14)<br>Pos (31)<br>NA (10)	G1-2 (13)<br>G3 (34)<br>NA (8)	Neg (25)<br>Pos (22)<br>NA (8)	Neg (28)<br>Pos (19)<br>NA (8)	Neg (4)<br>Pos (33)<br>NA (18)	NA (55)	NA (55)	49 [36-61]
Symmans (298)<br>GSE17705	A (298)	0 (227)<br>1 (71)	8.1<br>/ 10	1978-<br>2002	NA (298)	Neg (175)<br>Pos (112)<br>NA (11)	NA (298)	Pos (298)	NA (298)	NA (298)	yes (298)	no (298)	NA
Wang05 (286)<br>GSE2034	A (286)	0 (217)<br>1 (69)	NA<br>/ 5	1980-<br>1995	NA (286)	NA (286)	NA (286)	NA (286)	NA (286)	NA (286)	no (286)	no (286)	NA
Zhang (136)<br>GSE12093	A (136)	0 (116)<br>1 (20)	7.1<br>/ 10	1981-<br>2000	NA (136)	NA (136)	NA (136)	NA (136)	NA (136)	NA (136)	yes (136)	no (136)	NA
Zhou (54)<br>GSE7378	earlA (54)	0 (38)<br>1 (16)	5.3<br>/ NA	1989-<br>2004	NA (54)	Neg (54)	NA (54)	Pos (54)	NA (54)	NA (54)	NA (54)	NA (54)	44 [40-74]
3940 patients	earlA (54)<br>2+ (1310)<br>A (2090)<br>A+B (486)	0 (2809)<br>1 (1001)<br>NA (130)	5.7 yrs<br>(median)	1978-<br>2007	T0 (8) / T1 (744)<br>T2 (1329)<br>T3 (190) / T4 (61)<br>NA (1608)	Neg (1698)<br>Pos (1375)<br>NA (867)	G1-2 (1228)<br>G3 (826)<br>NA (1886)	Neg (692)<br>Pos (1832)<br>NA(1416)	Neg (473)<br>Pos (589)<br>NA (2878)	Neg (925)<br>Pos (203)<br>NA (2812)	no (1037)<br>yes (1522)<br>NA (1381)	no (2038)<br>yes (521)<br>NA (1381)	53 [45-62]

Supplementary Table S2: Merged Affymetrix cohort patient characteristics

Patients were included according to inclusion criteria from bc-GenExMiner v4.5 website: invasive carcinomas from female breast cancer, metastasis-free at diagnosis, with no neoadjuvant therapy before tumor collection and no FFPE samples. Tumor RNA extracted from fresh-frozen tumor macrodissection, except for Rody and Hatzis cohort (fresh-frozen biopsy). All types of treatment (no adjuvant therapy, radiotherapy or adjuvant therapy) were included. Treatments were rule-based and chosen by the medical teams on site according to patient clinical characteristics, physiological status and age, and IHC status for ER, PR and HER2. All types of recurrence (local, regional, distant) were included. "Reference ID" refers to identifiers from Gene Expression Omnibus (GEO) or the European Bioinformatics Institute (EBI) databases. Follow-ups are in years (yrs). "Q1" and "Q3" refer to lower quartile and upper quartile, respectively. "Pos" and "Neg" stand for positive and negative, and "NA" for not available. G1-2 refers to low grade and intermediate grade, and G3 to high grade. The four categories for tumor size are defined as: T0 (no evidence of tumor) < T1 ≤ 2 cm < T2 ≤ 5 cm < T3, and T4 means the tumor has grown into the neighboring tissue.

batch + reference ID	platform	relapse (or death)	follow-up median/end	time period	tumor size	lymph nodes	histological grade	ER	PR	HER2	hormono-therapy	chemo-therapy	median age [Q1-Q3]
Buffa (134)<br>GSE22219	Illumina humanRef-8 v1.0	0 (85)<br>1 (49)	10 yrs<br>/10	1989-1992	T0-1 (42)<br>T2-4 (92)	Neg (78)<br>Pos (56)	G1-2 (94)<br>G3 (27)<br>NA (13)	Pos (134)	NA (134)	NA (134)	yes (134)	no (120)<br>yes (14)	57 [49-64]
Chanrion (155)<br>GSE9893	MLRG Hum. 21K V12.0	0 (103)<br>1 (52)	5.1<br>/5	1989-2001	T0-1 (49)<br>T2-4 (101)<br>NA (5)	Pos (139)<br>NA (16)	G1-2 (115)<br>G3 (33)<br>NA (7)	Neg (8)<br>Pos (147)	Neg (29)<br>Pos (126)	NA (155)	yes (155)	no (155)	68 [60-75]
ICO TNBC (107)<br>GSE58812	Affymetrix HGU133 2+	0 (70)<br>1 (37)	6.7<br>/NA	1998-2007	T0-1 (53)<br>T2-4 (53)<br>NA (1)	Neg (77)<br>Pos (30)	G1-2 (16)<br>G3 (91)	Neg (107)	Neg (107)	Neg (107)	no (106)<br>yes (1)	no (12)<br>yes (95)	57 [49-66]
Juin (71)<br>GSE140489	Affymetrix HGU133 2+	0 (63)<br>1 (8)	4.3<br>/NA	2009-2016	T0-1 (24)<br>T2-4 (47)	Neg (28)<br>Pos (39)<br>NA (4)	G1-2 (40)<br>G3 (28)<br>NA (3)	Neg (10)<br>Pos (61)	Neg (17)<br>Pos (54)	Neg (69)<br>Pos (2)	no (10)<br>yes (61)	no (23)<br>yes (48)	64 [53-70]
METABRIC (1903)<br>brca_metabric	Illumina HT-12 v3	0 (1132)<br>1 (771)	8.4<br>/NA	1977-2005	T0-1 (591)<br>T2-4 (1292)<br>NA (20)	Neg (992)<br>Pos (911)	G1-2 (905)<br>G3 (926)<br>NA (72)	Neg (429)<br>Pos (1444)<br>NA (30)	Neg (895)<br>Pos (1008)	0 (1667)<br>1 (236)	0 (729)<br>1 (1174)	0 (1507)<br>1 (396)	62 [51-71]
Saal (3069)<br>GSE96058	Illumina HiSeq 2000	0 (2747)<br>1 (322)	4.5<br>/NA	2010-	T0-1 (1796)<br>T2-4 (1241)<br>NA (32)	Neg (1811)<br>Pos (1162)<br>NA (96)	G1-2 (1893)<br>G3 (1115)<br>NA (61)	Neg (224)<br>Pos (2646)<br>NA (199)	Neg (362)<br>Pos (2377)<br>NA (330)	Neg (2572)<br>Pos (392)<br>NA (105)	0 (681)<br>1 (2366)<br>NA (22)	0 (1807)<br>1 (1241)<br>NA (21)	64 [53-71]

Supplementary Table S3: Validation cohort patient characteristics

All types of recurrence (local, regional, distant) were included. "Reference ID" refers to identifiers from Gene Expression Omnibus (GEO) or cBioportal. Follow-ups are in years (yrs). Relapse was the clinical endpoint used for all datasets except for Saal dataset where death status was used."Q1" and "Q3" refer to lower quartile and upper quartile, respectively. "Pos" and "Neg" stand for positive and negative, and "NA" for not available. G1-2 refers to low grade and intermediate grade, and G3 to high grade. The four categories for tumor size are defined as: T0 (no evidence of tumor) < T1 ≤ 2 cm < T2 ≤ 5 cm < T3, and T4 means the tumor has grown into the neighboring tissue. Guedj dataset is presented in Supplementary Table 2 because it was initially included in the merged Affymetrix cohort for normalization (but it was not included in the training cohort during the ML analysis).

algo.	full name	R package	tested parameters	selected parameters for undersampled dataset
Enet	elastic net	*glmnet*	**alpha** = from 0 to 1, by 0.1<br>**lambda** = lambda.min<br>**variable =** 50, 400, 1000, 2000 or 5000	alpha = 0.1<br>lambda = lambda.min<br>variable = 5000
KNN	k-nearest neighbors	*rknn*	**k** = 3, 7, 13, 19 or 25<br>**variable =** 50, 400, 1000, 2000 or 5000	k = 13<br>variable = 1000
LR	logistic regression	*stats*	**variable =** 50, 400, 1000, 2000 or 5000<br>family = binomial(), maxit = 1000	variable = 50
Nnet	neural network	*caret / nnet*	**tuneGrid:**<br>• size = from 1 to 10, by = 1<br>• decay = from 10^{-2}, to 10^{3}, by 10^{1}<br>**variable =** 50, 400, 1000, 2000 or 5000	tuneGrid bestTune<br>variable = 400
RF	random forest	*randomForest*	**ntree** = 300, 500 or 1000<br>**mtry** = sqrt(p), sqrt(p)/2, sqrt(p)x2<br>**variable =** 50, 400, 1000, 2000 or 5000	ntree = 500<br>mtry = sqrt(p)<br>variable = 5000
SVM	support vector machine	*caret / kernlab*	**method** = svmLinear, svmRadial, svmPoly<br>**tuneGrid for svmRadial:**<br>• C = from 10^{-2}, to 10^{10}, by 10^{1}<br>• sigma = from 10^{-9}, to 10^{4}, by 10^{1}<br>**variable =** 50, 400, 1000, 2000 or 5000	method = svmRadial<br>tuneGrid bestTune<br>variable = 5000
VSURF	variable selection using RF	*VSURF*	**ntree** = 500<br>**variable** = 50, 400, 1000	ntree = 500<br>variable = 400
XGB	extreme gradient boosting	*caret / xgboost*	**tuneGrid:**<br>• nrounds = 50<br>• max_depth = 3, 6 and 9<br>• eta = 0.1, 0.01 and 0.001<br>• gamma = 0, 3 and 6<br>• colsample_bytree = 1<br>• min_child_weight = 1, 20, and 50<br>• subsample = 0.8<br>**variable =** 50, 400, 1000, 2000 or 5000	tuneGrid bestTune<br>variable = 2000

Supplementary Table S4: Algorithm parameter optimization

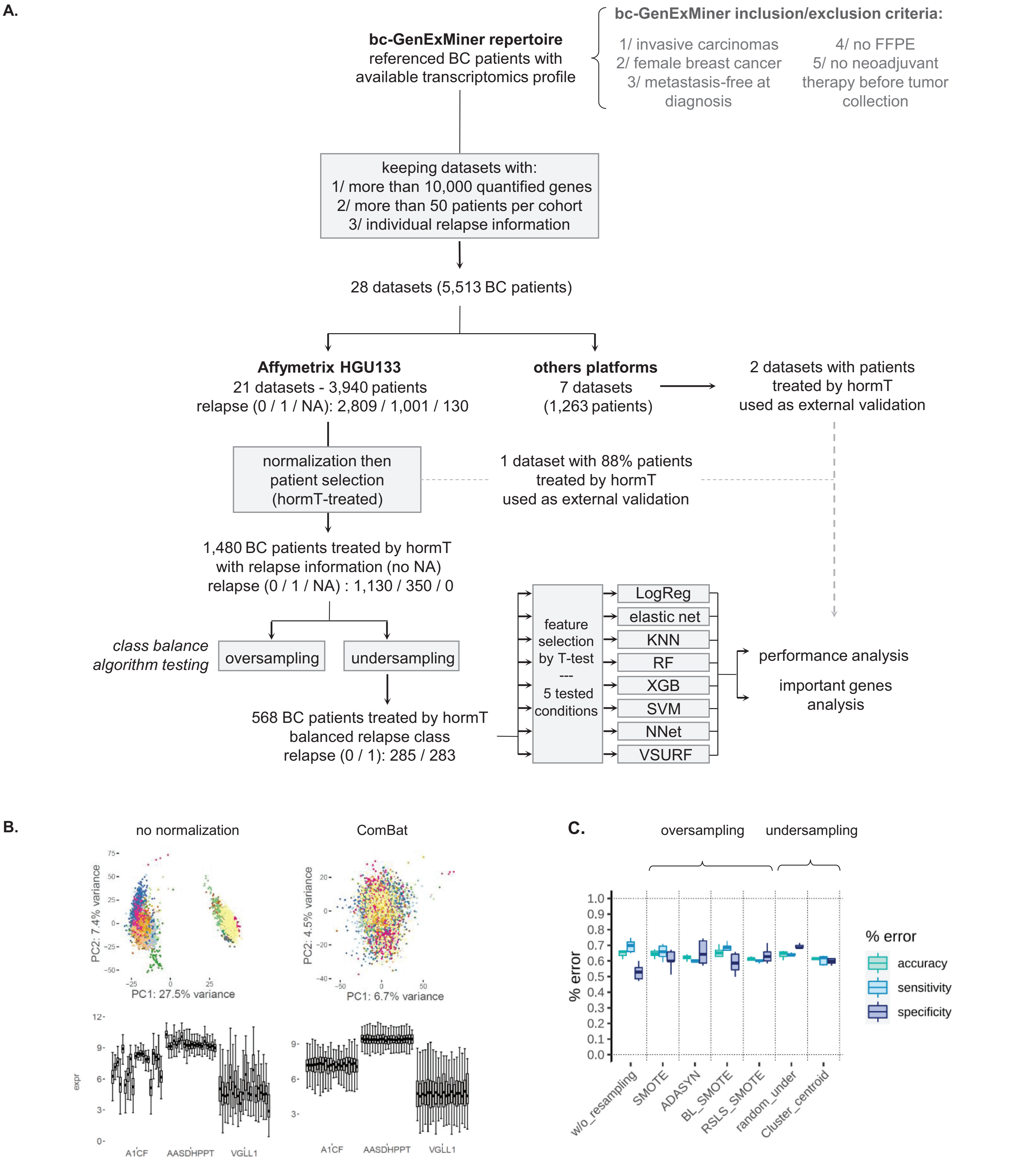

Supplementary Figure S1: Patient selection and algorithm pipeline configuration

A. Pipeline for breast cancer patient selection and machine learning analysis. "BC" stands for breast cancer, "hormT" for hormonotherapy, and "NA" for not available. Algorithms (abbreviated here) are fully described in Supplementary table 3. **B.** PCA was performed on merged transcriptomic datasets before and after cross-platform normalization using Combat (upper panels). Samples were colored according to batch (n=21). Three randomly picked genes were plotted according to each batch in the different normalizations (lower panels). **C.** Oversampling and undersampling methods were tested to obtain balanced class dataset, and the efficacy of resampling was estimated using random forest algorithm prediction on each class.

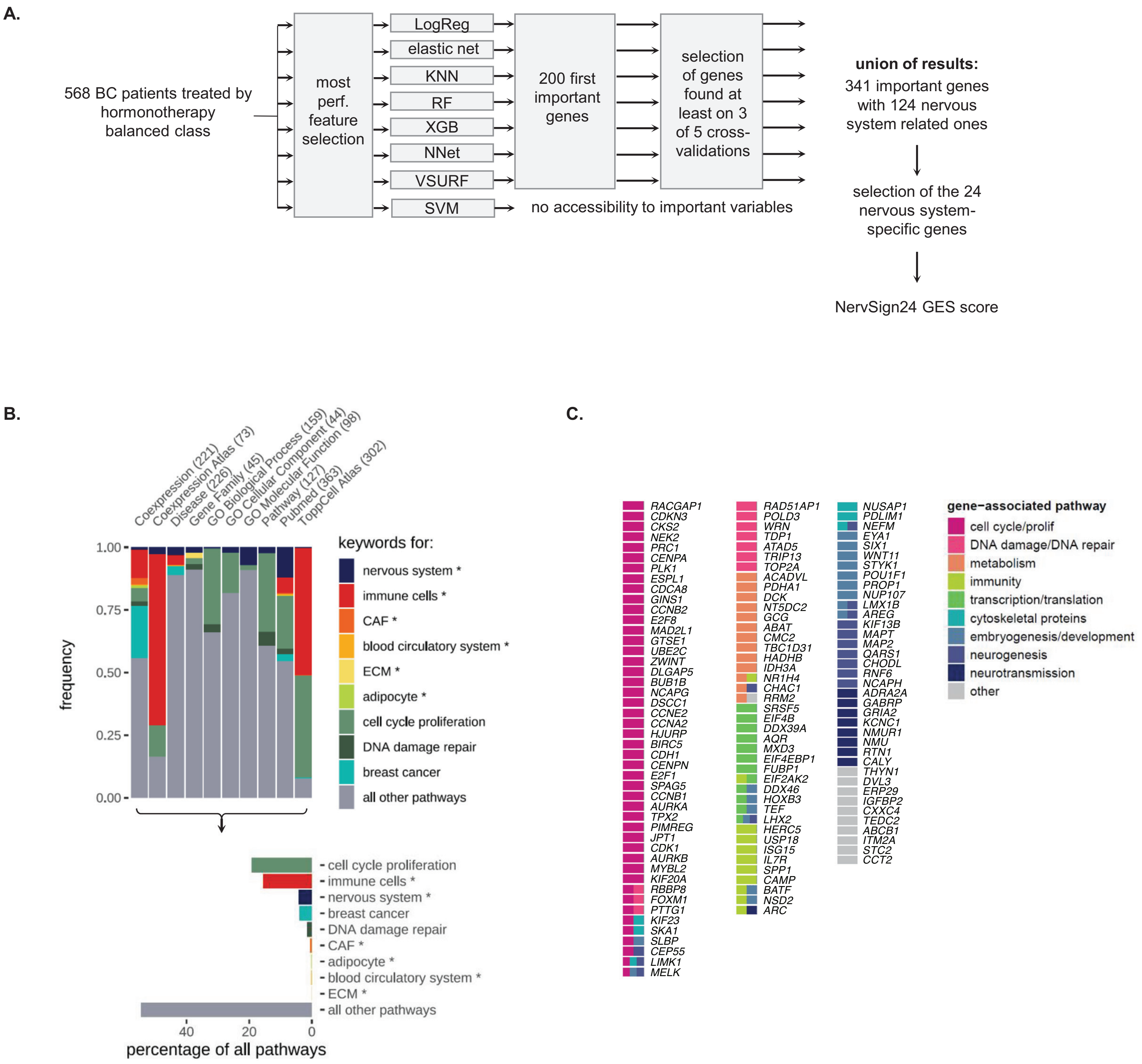

Supplementary Figure S2: Generation of NervSign24

A. Pipeline for machine learning analysis followed by biological selection of important variables leading to NervSign24 signature generation. **B.** ToppGene analysis was performed on the union of the 200 first important genes derived from seven machine learning models for relapse prediction in the 568-patient cohort, and statistically relevant associated pathways were listed for each ToppGene category. For each pathway, subcategories were then defined according to keyword search (or keyword abbreviation search) as following: nervous system: "*brain; myeli; oligod; glia; neur; axo; nerv; adren; noradr; epinephrine; acetylcholine; synap; trk; dopam; sensory*" - Immune cells: "*lymph; NK; T_cell; B_cell; dendrit; plasma; neutroph; monoc; immun; mast_cell; macropha; eosinoph*" - CAF: "*cancer-associated_fibroblast; CAF; fibroblast; FGF; -SMA*" - blood circulatory system: "*angiogenesis; blood_vessel; vascul; VEGF; Ang1; Ang2; Endothelial*" - ECM: "*extracellular; ECM; MMP; colla; fibronectin; laminin; tenascin; hyaluronic; heparin; versican; lumican; decorin*" - adipocyte: "*adipo; fat_tissue; fatty_tissue*" - cell cycle/prolif: "*cycle; M_phase; mitotic; anaphase; metaphase; mitose; G1; G2; division; prolif; divid; cyclin; aurora*" - DNA damage/repair: "*dna_damage; dna_integrity; dna_repair; strand_break; BRCA; HDR_; HRR; DSB; SDSA; _SSA*" - breast cancer: "*breast; mamm; MCF10*" The search was double-checked to remove false-positive hits. Numbers in parentheses in x-axe indicate the number of significant pathways per ToppGene category, and * indicates subcategories associated to TME (upper panel). The percentage of "pathway per defined subcategory over all elements" was plotted in lower panel with * indicating TME-related subcategories. **C.** Detailed gene list from the heatmap in Figure 2A (with the same order and categories than in Figure 2A).

A.

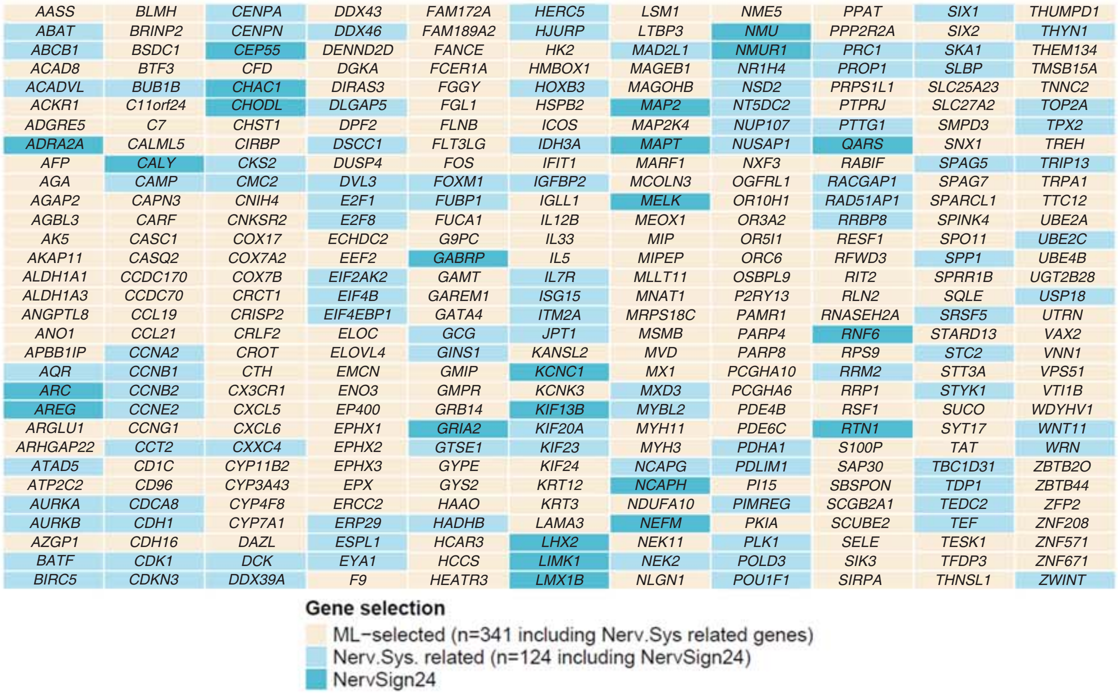

B.

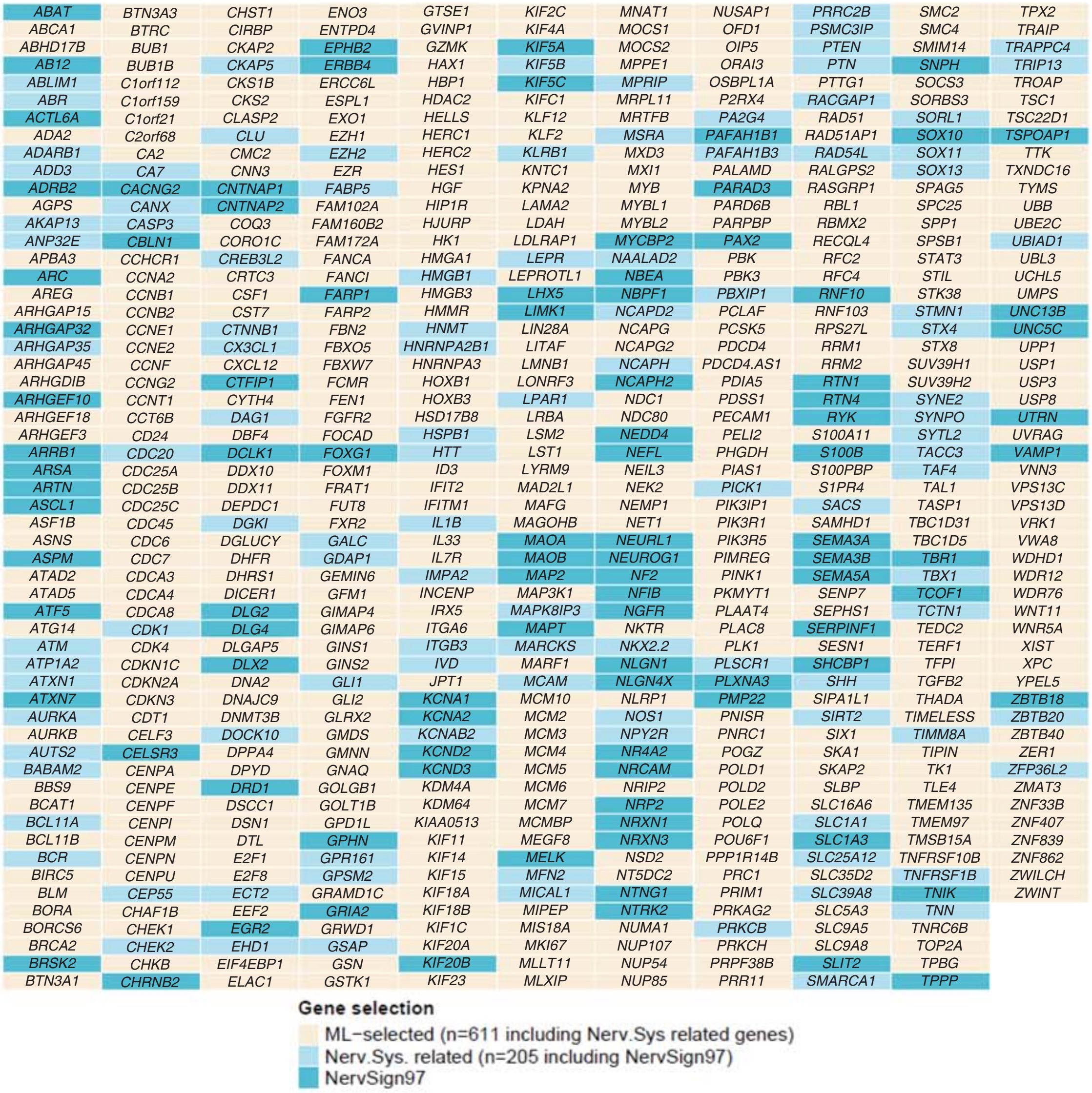

Supplementary Figure S3: ML-generated gene lists for the first signature and second signature generations

The genes obtained from the first ML analysis (see pipeline in Supplementary Figure 2A) and from the second ML analysis (see pipeline in Figure 2C) are presented **A.** and in **B.**, respectively. *ABAT* was initially not included in the first signature because its main role is associated with glutamate metabolism, but was finally retained in the second signature since it also plays a role in GABA neurotransmitter generation and could also be associated with the nervous system.

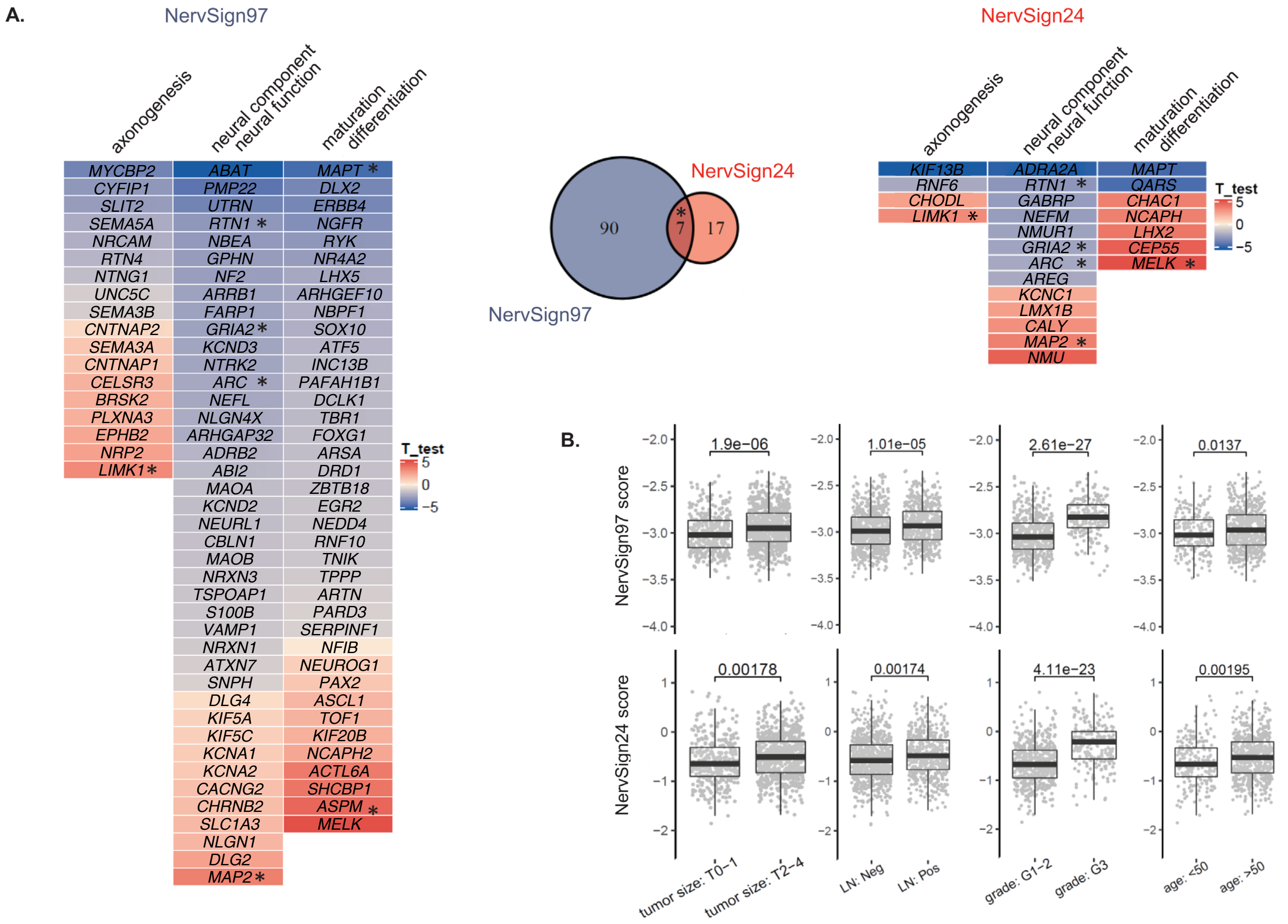

Supplementary Figure S4: Supplementary information related to Figure 2

A. Detailed gene list for NervSign97 and NervSign24. For both GES, genes were regrouped in three categories: axonogenesis-related, neural component/function- related, and neural maturation/differentiation- related. Genes were colored according to T-test statistic between relapse/no relapse class in the 568-patient training cohort. T-test statistic was used as direction indicator for the calculation of weighted average expression. Asterisks indicate the common genes between the two signatures. **B.** Box plot of Nervsign24 and NervSign97 weighted average expression score according to prognostic variables (tumor size, axillary lymph node (LN) status, histological grade and age) in hormonotherapy-treated patients (unbalanced merged Affymetrix cohort, n=1480). Statistical significance between groups was assessed using unpaired two-tailed Student's T-test.

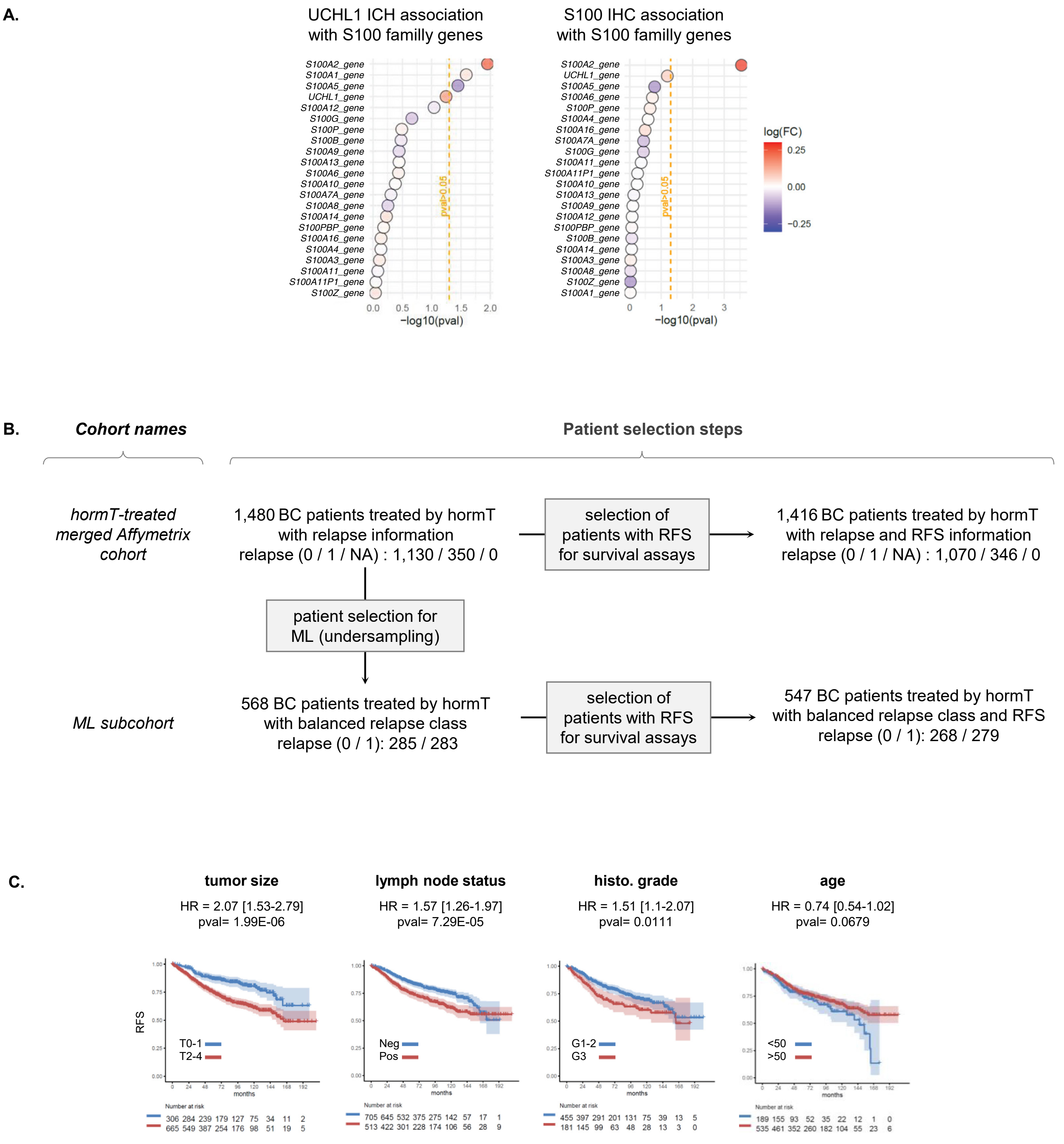

Supplementary Figure S5: Supplementary information related to Figure 3

A. Association of S100 family gene and UCHL1 gene with IHC nervous markers status (UCHL1 and S100) in 107-patient TNBC cohort (n=85 and n=80, respectively). **B.** Description of the cohorts used in machine learning (ML) analyses and survival analyses. "BC" stands for breast cancer, "hormT" for hormonotherapy, and "NA" for not available. **C.** RFS Kaplan-Meier analysis was performed in patient cohort stratified according to prognostic variables in hormonotherapy-treated patients (unbalanced merged Affymetrix cohort, n=1416). The shaded area represents the 95% CI. The four categories for tumor size are defined as: T0 (no evidence of tumor) < T1 ≤ 2 cm < T2 ≤ 5 cm < T3 < T4 (the tumor has grown into the neighboring tissue). G1-2 refers to low grade and intermediate grade, and G3 to high grade.

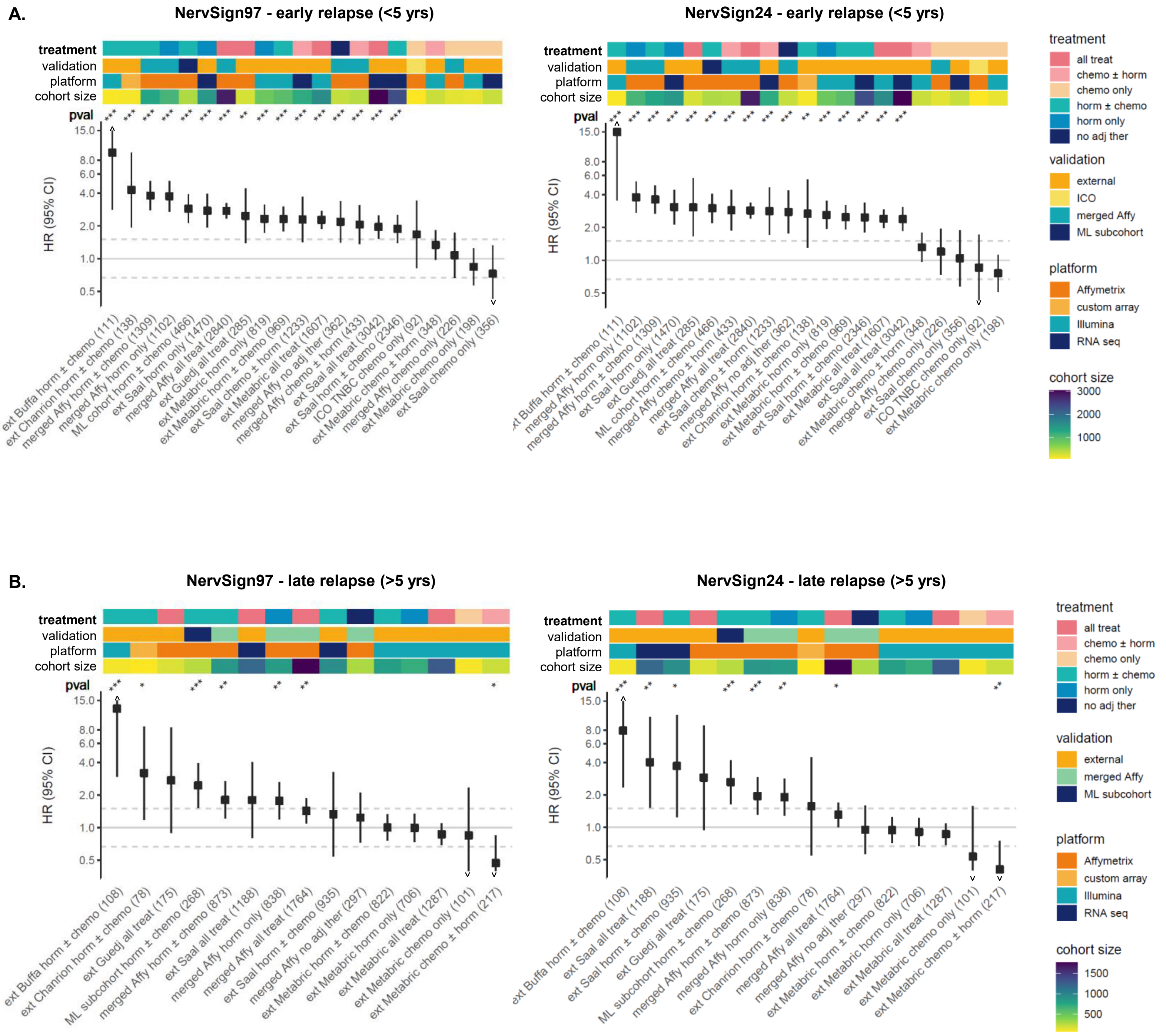

Supplementary Figure S6: Univariate Cox analyses in cohorts of patients with early or late recurrence

A. and B. Hazard ratios (HR), confidence intervals (CI) and associated *p*-values (pval) were calculated using univariate Cox analysis in various cohorts of patients, with early (**A.**) or late (**B.**) relapse, and stratified according to NervSign97 or NervSign24 median score. All HR were related to RFS except for Saal cohort (OS). ***, ** and * stand for for *p*-value <0.001, 0.01 and 0.5, respectively. "horm ± chemo" and "chemo ± horm" refer to patients treated with hormonotherapy or chemotherapy with no exclusivity, whereas "horm only" and "chemo only" indicate exclusivity. "Ext" refers to external validation cohorts, "ICO" to our hospital validation cohort, "merged Affy" to the 3,940-patient Affymetrix cohort, and "ML subcohort" to the subset population used to create ML derived-gene signatures. Numbers between parentheses indicate cohort size.

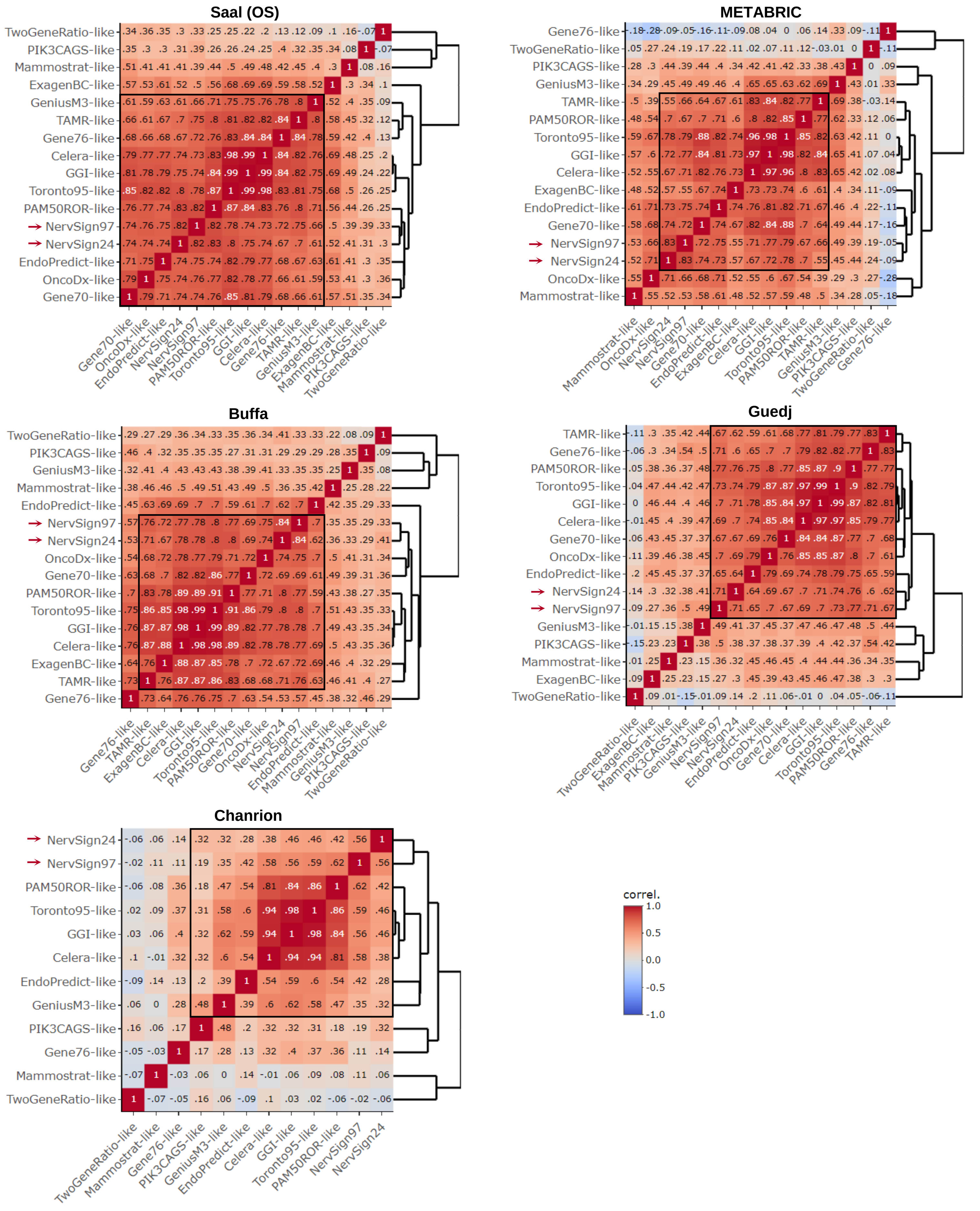

Supplementary Figure S7: Supplementary information related to Figure 4

Pearson correlation between the 16 signatures scores (or 12 for Chanrion cohort) were hierarchically clustered and plotted accordingly in four independent cohorts (hormonotherapy-treated patients only). The most related signatures were identified using the dendrogram and highlighted with a black square box.

A.

merged Affymetrix (RFS)

NervSign97

label	HR (95% CI)	P	
NervSign97	1.57 (1.34−1.84)	2.7e−08	***
age >50	0.59 (0.4−0.87)	0.0075	**
chemo	0.83 (0.53−1.32)	0.44	
tumor size: T2−4	1.75 (1.16−2.64)	0.0075	**
LN: Pos	1.33 (0.95−1.85)	0.095	
grade: G3	0.91 (0.64−1.3)	0.6	

NervSign24

label	HR (95% CI)	P	
NervSign24	1.62 (1.38−1.91)	7.8e−09	***
age >50	0.59 (0.4−0.86)	0.0062	**
chemo	0.89 (0.56−1.42)	0.63	
tumor size: T2−4	1.83 (1.22−2.76)	0.0035	**
LN: Pos	1.46 (1.05−2.04)	0.025	*
grade: G3	0.85 (0.59−1.23)	0.39	

METABRIC (RFS)

NervSign97

label	HR (95% CI)	P	
NervSign97	1.3 (1.15−1.42)	4.5e−06	***
age >50	1.1 (0.82−1.51)	0.51	
chemo: Pos	1.2 (0.95−1.61)	0.11	
tumor size: T2−4	1.3 (1−1.58)	0.045	*
LN: Pos	1.9 (1.51−2.31)	7.9e−09	***
grade: G3	1.1 (0.91−1.4)	0.27	

NervSign24

label	HR (95% CI)	P	
NervSign24	1.3 (1.15−1.42)	7.3e−06	***
age >50	1.1 (0.83−1.53)	0.45	
chemo: Pos	1.2 (0.91−1.55)	0.2	
tumor size: T2−4	1.3 (1.03−1.62)	0.024	*
LN: Pos	1.9 (1.53−2.35)	3.5e−09	***
grade: G3	1.1 (0.92−1.41)	0.23	

Saal (OS)

NervSign97

label	HR (95% CI)	P	
NervSign97	1.34 (1.14−1.58)	0.00055	***
age >50	3.56 (1.64−7.75)	0.0014	**
chemo: Pos	0.31 (0.21−0.46)	5.6e−09	***
tumor size: T2−4	2 (1.47−2.72)	1.1e−05	***
LN: Pos	1.4 (1.05−1.89)	0.023	*
grade: G3	1.57 (1.1−2.24)	0.013	*

NervSign24

label	HR (95% CI)	P	
NervSign24	1.49 (1.26−1.75)	1.8e−06	***
age >50	3.38 (1.55−7.36)	0.0022	**
chemo: Pos	0.31 (0.21−0.45)	2.9e−09	***
tumor size: T2−4	1.97 (1.45−2.68)	1.6e−05	***
LN: Pos	1.42 (1.06−1.91)	0.019	*
grade: G3	1.43 (1.01−2.02)	0.044	*

B.

METABRIC (RFS)

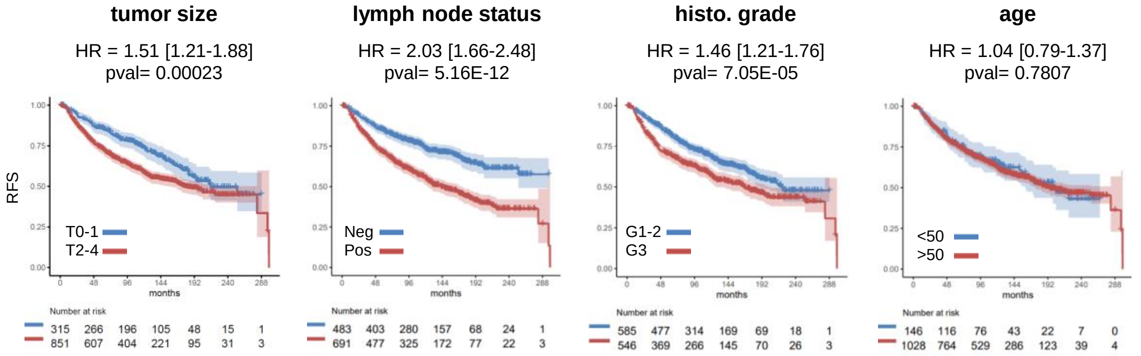

Saal (OS)

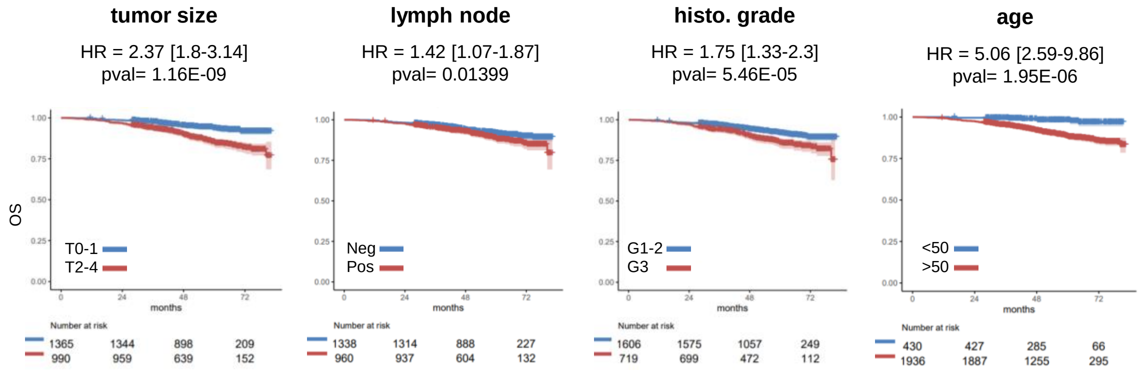

Supplementary Figure S8: Multivariable and univariate Cox analyses in merged Affymetrix, METABRIC and Saal cohorts

A. Multivariate Cox proportional regression analyses for RFS (merged Affymetrix and METABRIC cohorts) and OS (Saal cohort) considering nervous signature z-score, age, chemotherapy status, tumor size (T0-1 < 2 cm < T2-4), lymph node status (negative or positive) and histological grade (G1+G2 versus G3). **B.** Univariate Kaplan-Meier survival analysis in patients stratified by tumor size, lymph node status, histological grade or age (METABRIC and Saal cohorts).

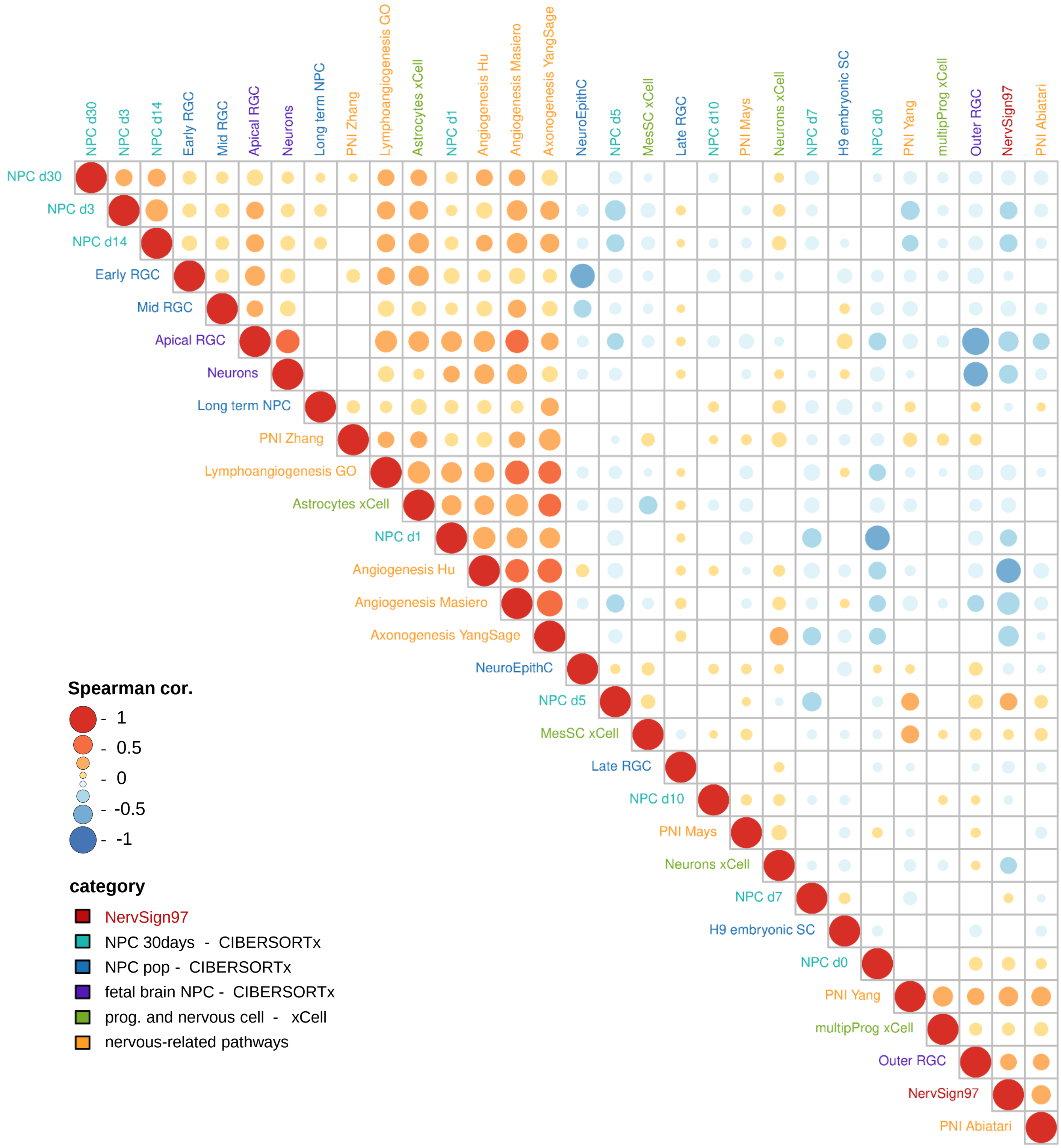

Supplementary Figure S9: Correlation between nervous system mechanisms and NervSign97

Correlation matrix combining CIBERSORTx abundance scores, xCell abundance score, nervous/regeneration pathway enrichment scores, and NervSign97 score in the hormonotherapy treated merged Affymetrix cohort (n= 1,416).

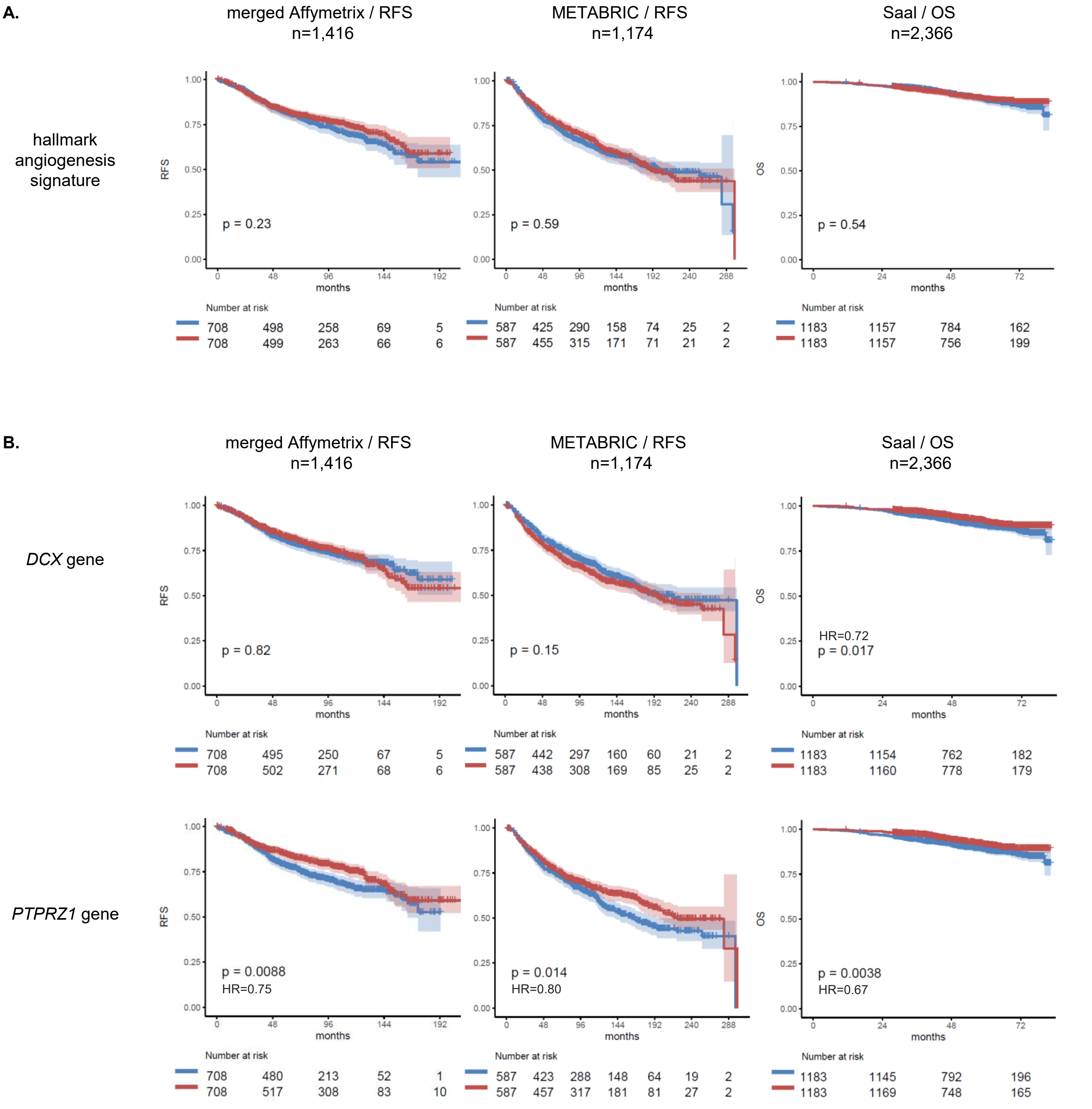

Supplementary Figure S10: Kaplan-Meier analysis of previously published biomarkers

RFS or OS Kaplan-Meier analyses were performed in three hormonotherapy-treated patient cohorts stratified according to "hallmark angiogenesis" GSVA score median (**A.**), or *DCX* and *PTPRZ1* expression medians (**B.**). The shaded area represents the 95% confidence intervals. The *p*-value was derived from a log-rank test comparing the two groups. HRs from Cox model are indicated when *p*-value is significant.
